# Shenling Baizhu San attenuates testicular spermatogenic dysfunction in hyperuricemic mice via dual modulation of MAPK/NF-κB and NLRP3 inflammasome pathways

**DOI:** 10.1186/s41065-025-00553-x

**Published:** 2025-09-29

**Authors:** Xiaocui Jiang, Zhongyi Zhu, Qi Liu, Xingyu Jiang, Zihao Liu, Shuang Li, Jigang Cao, Min Xiao

**Affiliations:** 1 Laboratory Animal Research Center, Hubei University of Chinese Medicine, Wuhan, China; 2 Hubei Shizhen Laboratory, Wuhan, China; 3 College of Traditional Chinese Medicine, Hubei University of Chinese Medicine, Wuhan, China; 4 Department of Urology, Hubei Provincial Hospital of Traditional Chinese Medicine, Wuhan, China; 5 School of Basic Medical Sciences, Hubei University of Chinese Medicine, Wuhan, China

**Keywords:** Shenling baizhu san, Spermatogenic dysfunction, Hyperuricemia, MAPK/NF-κB signaling pathway, NLRP3 inflammasome pathway

## Abstract

**Context:**

Hyperuricemia (HUA) is a known factor contributing to testicular spermatogenic dysfunction. Shenling Baizhu San (SLBZS), a traditional Chinese medicine compound, has demonstrated efficacy in reducing uric acid levels. However, its specific impact on testicular spermatogenic function in mice with HUA remains unclear.

**Objective:**

To investigate the impact and mechanism of SLBZS on testicular spermatogenic function in HUA mice.

**Materials and methods:**

A hyperuricemia-induced spermatogenic dysfunction model was created by administering intraperitoneal injections of potassium oxonate (600 mg/kg/d) for seven days. Following model establishment, 48 Balb/c mice were randomly divided into six groups: control, model, low-dose SLBZS (5.04 g/kg/d), medium-dose SLBZS (10.07 g/kg/d), high-dose SLBZS (20.14 g/kg/d), and febuxostat (10 mg/kg/d). All groups, except the control, underwent model induction, followed by specific interventions. Subsequent analyses included serum uric acid levels, testicular and epididymal indices, histopathological assessments, sperm quality, oxidative stress and inflammation markers, and the expression of proteins related to apoptosis and inflammation signaling pathways.

**Results:**

SLBZS markedly enhanced sperm quality, testicular and epididymal indices, and serum uric acid levels in mice, while ameliorating histopathological lesions in testicular tissue. Additionally, SLBZS significantly reduced oxidative stress, serum inflammation markers, and testicular cell apoptosis, with the high-dose group showing superior effects compared to the febuxostat group. Further investigation revealed that SLBZS inhibited the expression and phosphorylation of proteins in the MAPK/NF-κB pathway and suppressed the expression of proteins in the NLRP3 inflammasome pathway.

**Discussion and conclusions:**

SLBZS potentially modulates the MAPK/NF-κB and NLRP3 inflammasome signaling pathways, thereby suppressing inflammatory responses and enhancing spermatogenesis in the testes of HUA mice.

**Supplementary Information:**

The online version contains supplementary material available at 10.1186/s41065-025-00553-x.

## Introduction

Testicular spermatogenic dysfunction arises from impaired spermatogenic cells, reducing spermatogenic activity in the seminiferous epithelium and causing conditions like oligospermia, asthenospermia, or azoospermia, which severely affect male fertility. The prevalence and significance of testicular spermatogenic dysfunction are often underestimated, accounting for approximately 75% of male infertility cases globally [1]. In addition to affecting male sexual function and fertility, it can manifest systemic symptoms like sleep disturbances and osteoporosis [2, 3]. Furthermore, it is closely linked to the development of chronic conditions such as diabetes, cardiovascular diseases, and cerebrovascular diseases, thereby influencing male health and overall quality of life [4, 5]. The condition is multifactorial, encompassing genetic predisposition, endocrine disorders, varicocele, lifestyle factors, and environmental exposures. Recent research has identified hyperuricemia (HUA) as a contributing factor to spermatogenic dysfunction [6]. Clinically, HUA is frequently observed in individuals with metabolic syndrome, obesity, and renal dysfunction, as well as in patients receiving certain medications such as diuretics or immunosuppressants. Given the rising prevalence of HUA, particularly among younger individuals, its impact on male reproductive health is gaining clinical recognition. Lowering serum uric acid levels to alleviate spermatogenic dysfunction induced by HUA represents a promising strategy for preventing male infertility at its root cause.

HUA disrupts mitochondrial sodium-calcium exchange, enhances superoxide anion production, and impairs the antioxidant system, leading to increased reactive oxygen species (ROS) levels and oxidative stress (OS) [7]. This disruption causes an imbalance in the oxidation and antioxidation processes in the body, leading to chronic inflammatory damage [8]. Stimulation of cell surface receptors by OS induces pro-inflammatory responses, leading to the release of multiple cytokines and the accumulation of small intracellular molecules, thereby enhancing the inflammatory reaction. Research has shown that inflammation can disturb the blood-testis barrier (BTB) by affecting cell junction proteins [9]. The excessive release of inflammatory mediators can impede phagocytic function, trigger an inflammatory innate immune response, and ultimately induce cell apoptosis [10]. This process gradually harms cells involved in sperm production and support cells in the testicular tubes, resulting in the total impairment of sperm production and a decrease in male fertility.

The Mitogen-activated protein kinase (MAPK)/nuclear factor κB (NF-κB) pathway is an important inflammatory pathway [11]. OS can stimulate MAPK, initiating NF-κB activation, resulting in the secretion of inflammatory agents like iNOS, IL-6, IL-1β, COX-2, and TNF-α [12]. Nucleotide-binding oligomerization domain-like receptor protein 3 (NLRP3)/cysteine-requiring aspartate protease-1 (Caspase-1)/IL-1β is a typical inflammasome pathway [13]. The activation of NLRP3 inflammasome will also promote the secretion of inflammatory mediators such as IL-1β and IL-18 [14]. Research indicates that inhibition of the MAPK/NF-κB and NLRP3 inflammasome pathways can suppress the release of inflammatory mediators, thereby reducing inflammation, lowering cell apoptosis, and mitigating damage to spermatogenic and Sertoli cells in seminiferous tubules. Consequently, we speculate that the enhancement of testicular spermatogenic function in HUA mice through uric acid reduction may involve the regulation of the MAPK/NF-κB and NLRP3 inflammasome pathways, thereby inhibiting the inflammatory response induced by OS.

Traditional Chinese medicine (TCM) offers alternative therapeutic approaches, with proven efficacy in reducing uric acid levels [15]. SLBZS is a TCM formula aimed at lowering uric acid levels by enhancing spleen function and eliminating dampness. This formula was initially documented in the ancient TCM text “Taiping Huimin Heji Jufang”. Previous research has demonstrated the significant reduction of serum uric acid levels and enhancement of testicular spermatogenic function in hyperuricemia-induced mice [16]. However, the precise mechanism of action requires further investigation. In this study, we established a hyperuricemia-induced spermatogenic dysfunction mouse model to assess the impact of SLBZS on testicular spermatogenic function in hyperuricemia-afflicted mice. Additionally, we examined the involvement and modulation of the MAPK/NF-κB and NLRP3 inflammasome pathways, and assessed the anti-inflammatory properties of SLBZS in enhancing testicular spermatogenic function in hyperuricemia-induced mice. These findings may offer novel insights for the management and treatment of male infertility.

## Materials and methods

### Drugs, reagents and antibodies

SLBZS (100 g each of ginseng, stir-fried Atractylodes macrocephala, Poria cocos, Chinese yam, and licorice; 75 g of stir-fried white lentils; 50 g each of lotus seeds, stir-fried coix seeds, Amomum villosum, and Platycodon grandiflorum). The decoction components were sourced from the Hubei Provincial Hospital of Traditional Chinese Medicine and processed into a 2 g/mL concentration decoction at the hospital’s facility. Potassium oxonate (S17112-5 g) was obtained from Shanghai Yuanye Biotechnology Co., Ltd., while Febuxostat (H20130058) was acquired from Jiangsu Wanbang Biopharmaceutical Group Co., Ltd. The uric acid kit (C012-1-1) was purchased from Nanjing Jiancheng Bioengineering Institute. Assay kits for malondialdehyde (MDA) content (BC0025) and superoxide dismutase (SOD) activity (BC0170), along with the hematoxylin-eosin (H&E) staining kit (G1120), were procured from Beijing Solarbio Science & Technology Co., Ltd. Additionally, IL-6 (SEKM-0007), TNF-α (SEKM-0034), and IL-1β (SEKM-0002) ELISA kits were obtained from the same supplier. Rabbit monoclonal antibodies for Bcl-2 (A19693), Bax (A19684), β-actin (AC038), NF-κB p65 (A22331), iNOS (A3774), COX-2 (A3560), P-JNK (AP0631), P-P38 (AP1508), ERK (A19630), P-ERK (AP0974), NLRP3 (A24294), GSDMD (A20728), IL-1β (A23484), IL-6 (A22222), TNF-α (A22227) and ASC (A22046) were purchased from Wuhan Abiocenter Biotechnology Co., Ltd. Rabbit polyclonal antibodies for JNK (A0288), P38 (A14401), and Caspase-1 (A0964) were also sourced from Wuhan Abiocenter Biotechnology Co., Ltd. The horseradish peroxidase (HRP)-labeled goat anti-rabbit secondary antibody (AS014) was acquired from the same company. The TUNEL apoptosis detection kit (G1502-50T) was purchased from Wuhan Servicebio Technology Co., Ltd.

### Instrument

KZ-III-F high-speed low-temperature tissue grinder (Wuhan Servicebio Technology Co., Ltd.); CR21G cryogenic high-speed centrifuge (Hitachi, Japan); DYCZ-40 electroporator (Beijing Liuyi Instrument Factory); WLJY-9000 Weili color sperm quality detection system (Beijing Weili Technology Company); E100 microscope, NikonDS-U3 imaging system (Nikon Corporation, Japan); RM2016 pathological microtome (Shanghai Leica Instruments Co., Ltd.); JB-P5 embedding machine (Wuhan Junjie Electronic Co., Ltd.); Multiskan FC microplate reader, Q Exactive high-resolution mass spectrometer, and UltiMate 3000 RS chromatograph (Thermo Fisher Scientific (China) Co., Ltd.).

### Q-Orbitrap high-resolution liquid chromatography-mass spectrometry analysis of SLBZS

A high-resolution liquid chromatography-mass spectrometry (HR-LC-MS) system with a Q-Orbitrap was employed to identify chemical components in traditional Chinese medicine. The mass spectrometry parameters included an electrospray ionization (ESI) source with both positive and negative ion modes. The system operated in full scan/data-dependent MS2 scan mode, with resolutions of 70,000 for full scan and 17,500 for MS2 scan, covering a range of 100 to 1,500 m/z. The electrospray voltage was ± 3.2 kV, and the capillary was maintained at 300 °C. High-purity argon (≥ 99.999%) served as the collision gas, with normalized collision energies of 30, 40, and 60. Nitrogen (≥ 99.999%) was used as the sheath gas at 40 Arb and as the auxiliary gas at 15 Arb and 350 °C. Data acquisition spanned 30 min. Chromatographic conditions were specified as follows: a Welch AQ-C18 column (150 × 2.1 mm, 1.8 μm) with a flow rate of 0.30 mL/min. The aqueous phase contained 0.1% formic acid, and methanol served as the organic phase. The column oven was set to 35 °C, the autosampler to 10 °C, and the injection volume was 5.00 µL [17].

### Network Pharmacology

Active components of traditional Chinese medicine were identified via TCMSP using criteria of OB ≥ 30% and DL ≥ 0.18. Potential targets were refined through Uniprot calibration and removal of invalid entries. The GeneCards database was queried with keywords related to oligoasthenospermia to identify relevant targets. After deduplication and Uniprot verification, the disease target genes were confirmed. Drug targets were then compared with disease targets, and a Venn diagram identified intersecting genes. A “drug-component-action target” network was constructed using Cytoscape 3.8.2. These intersecting genes were uploaded to the String database to create a PPI network, which was analyzed and visualized with Cytoscape 3.8.2. The intersection genes underwent GO and KEGG pathway enrichment analysis via the DAVID database. Key entries were selected to predict the mechanism of traditional Chinese medicine in treating oligoasthenospermia [18].

### Animals

Eight-week-old SPF male Balb/c mice (*n* = 64, 18–22 g) were obtained from the Hubei Provincial Laboratory Animal Research Center and housed at the SPF-level facility of Hubei University of Chinese Medicine (Animal use permit: SYXK(E)2023-0067). The environment was controlled at 22 ± 2 °C, with 50%-70% relative humidity and a 12-hour light/dark cycle. Mice had ad libitum access to food and water. Following a 7-day acclimation, experiments commenced. All procedures adhered to the Guide for the Humane Use and Care of Laboratory Animals in Biomedical Research and were approved by the Animal Experiment Ethics Committee of Hubei University of Chinese Medicine (Approval No.: HUCMS83374166).

### Animal grouping, modeling and drug administration

After one week of adaptive feeding, the mice were categorized using the following protocol:

In the initial phase of the study, 16 male Balb/c mice were randomly assigned to either a control group or a model group, with each group comprising 8 mice. The model group underwent induction of hyperuricemia-induced testicular spermatogenic dysfunction according to references [19, 20] by receiving intraperitoneal injections of potassium oxonate suspension at a dosage of 600 mg/(kg·d) for 7 consecutive days. The control group was given daily intraperitoneal injections of normal saline at an equivalent volume for the same duration. After the 7-day period, serum uric acid levels, testicular index, epididymal index, and sperm quality were assessed to verify the successful establishment of the experimental model.

**Fig. 1:**
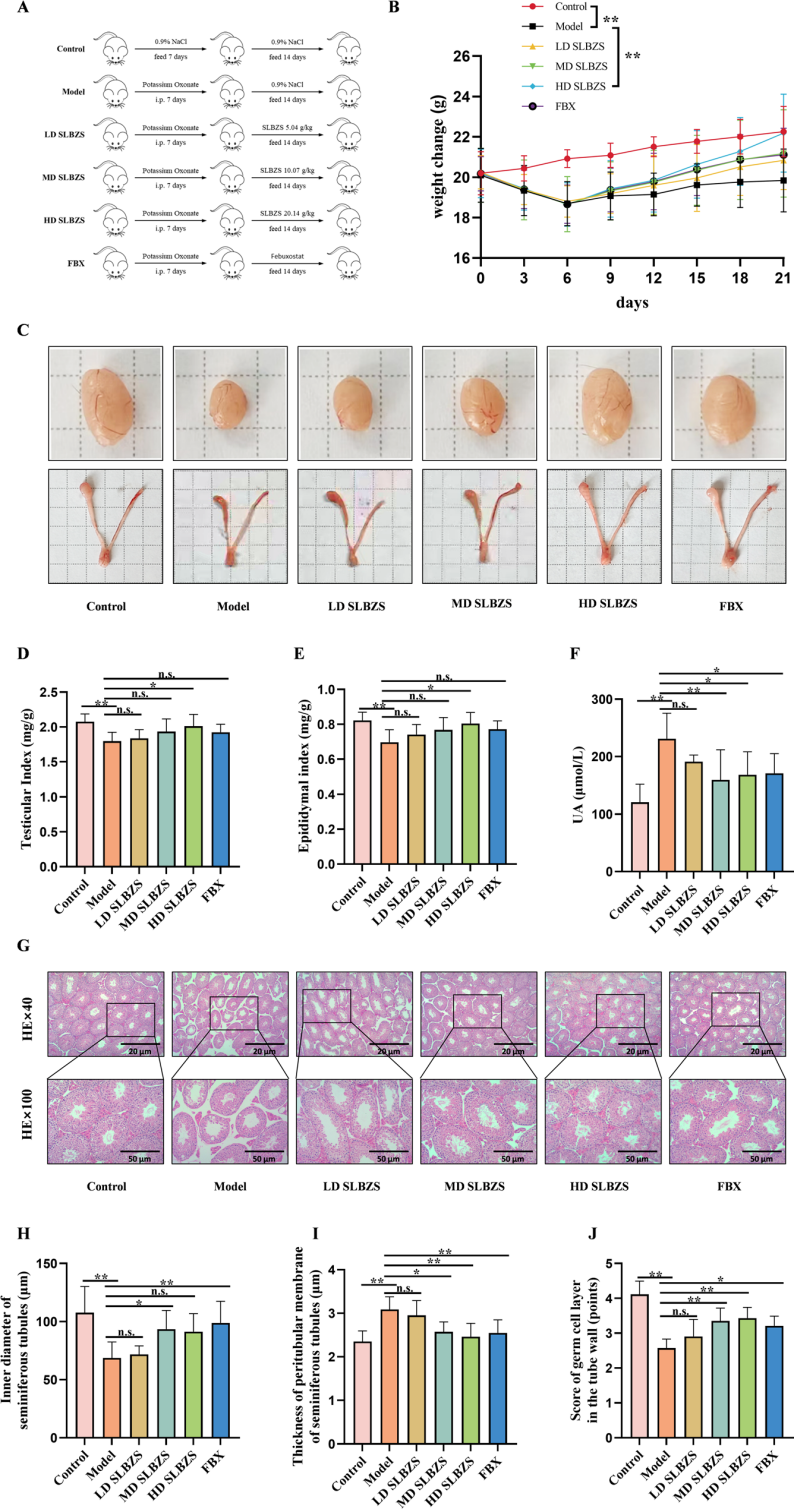
SLBZS treatment alleviated the histopathological lesions of testes in a potassium oxonate-induced HUA spermatogenic dysfunction mouse model. (**A**) Schematic diagram of modeling. (**B**) Changes in body weight of mice. (**C**) Pictures of testes and epididymis of mice. (**D**) Testicular index. (**E**) Epididymal index. (**F**) Serum UA content. (**G**) Representative H&E staining of testicular tissue in each group (**H**) Inner diameter of seminiferous tubules. (**I**) Thickness of peritubular membrane of seminiferous tubules. (**J**) Score of germ cell layer in the tube wall. Data are shown as the means ± SEM. ^*^p < 0.05; ^**^p < 0.01

In the second stage of the experiment, the remaining 48 male Balb/c mice were randomly assigned to six groups: control, model, high-dose SLBZS, medium-dose SLBZS, low-dose SLBZS, and febuxostat, with each group consisting of 8 mice. All groups, except for the control group, underwent the established modeling procedure. The control group received daily intraperitoneal injections of normal saline for 7 days. The modeling process was illustrated in Fig. 1A. Following modeling, and based on the conversion formula for body surface area between mice and humans, the medium dose of SLBZS was calculated to be 9.1 times the recommended adult dose. Specifically, the medium dose was calculated as follows: 77.5 g/70 kg × 9.1 = 10.07 g/kg. The high-dose group was given twice the medium dose, while the low-dose group received half the medium dose. The high-dose, medium-dose, and low-dose SLBZS groups were administered 20.14 g/kg/d, 10.07 g/kg/d, and 5.04 g/kg/d of SLBZS, respectively, via gavage with normal saline to adjust the volume. The febuxostat group received 10 mg/kg/d of febuxostat dissolved in normal saline by gavage. Febuxostat is a selective xanthine oxidase inhibitor clinically used for the treatment of hyperuricemia and gout, acting through inhibition of xanthine oxidase to reduce the synthesis of uric acid in vivo. In this study, febuxostat served as a positive control to validate the established hyperuricemia model and to provide a reference for comparing the therapeutic effects of SLBZS. The control and model groups were gavaged with normal saline at the same volume. Gavage was performed once daily for 14 consecutive days, and mouse body weight was recorded every three days. After mice were sacrificed at the end of the experimental period, both testes and epididymides were immediately dissected out, carefully trimmed of surrounding fat and connective tissue, and weighed using an electronic analytical balance (accuracy 0.01 g). The testicular index and epididymal index were calculated according to the following formulas: testicular index∉=∉[testes weight (g) / body weight (g)] × 100%; Epididymal index∉=∉[epididymides weight (g) / body weight (g)] × 100%.

### Content of UA in mouse serum

Following the final administration, the mice were fasted for 12 h without water deprivation. Anesthesia was induced via intraperitoneal injection of 1% pentobarbital sodium at a dosage of 50 mg/kg. Blood samples were obtained by enucleating the eyeballs and were collected in anticoagulant blood collection tubes. The samples were then left at room temperature for 1 h before being centrifuged at 300 r/min, with a centrifugal radius of 13 cm, for 15 min at 4 °C. The resulting supernatant was stored at 4 °C in a refrigerator for future use. Serum uric acid levels in the mice were determined using a UA kit.

### Hematoxylin-eosin (H&E) staining and histopathological analysis

After blood collection, the mice were euthanized via cervical dislocation and immediately placed on ice. The abdominal cavity was exposed promptly, and the testes were swiftly dissected. Their mass was recorded, and the testes were rinsed with normal saline before being immersed in testicular fixative for fixation at room temperature for 48 h. Subsequently, the testes were fixed, processed, and embedded in paraffin. Sections were prepared and subjected to hematoxylin-eosin (H&E) staining according to standard protocols for histopathological analysis.

Following sectioning, the samples were observed and photographed under an optical microscope. For each specimen, 10 cross-sections of seminiferous tubules were randomly selected under the microscope. The inner diameter (D) and the thickness of the peritubular membrane (M) of the seminiferous tubules were measured, and the average values were calculated. In addition, the number of layers of germ cells on the tubular wall § was assessed. The following scoring criteria were applied: 0 layers (0 points), 0 layers < *P* ≤ 1 layer (1 point), 1 layer < *P* ≤ 2 layers (2 points), 2 layers < *P* ≤ 3 layers (3 points), 3 layers < *P* ≤ 4 layers (4 points), and *P* > 4 layers (5 points).

### Sperm density and motility rate

The bilateral epididymis of each mouse was isolated and its mass was recorded. The epididymal tail was immersed in 1 mL of phosphate-buffered saline (PBS) preheated to 37 °C. The tissue was minced with ophthalmic scissors to release the sperm and was incubated at 37 °C for 15 min. The mixture was filtered through a 200-mesh sieve to separate the sperm from tissue fragments. The filtrate was gently mixed to obtain a sperm suspension. Sperm density and motility rate were analyzed using an automatic sperm analyzer.

### Sperm aniline blue staining

A volume of 15 µL of sperm suspension was spread on an anti-peeling slide and was allowed to air dry. The sample was fixed at room temperature for 15 min and then rinsed for 5 min. The slide was stained with aniline blue for 5 min at room temperature, followed by another 5-minute rinse. The stained smear was examined under a microscope. Mature sperm heads, which were lightly stained or unstained, indicated DNA tightly bound to protamine, reflecting high DNA maturity. In contrast, immature sperm heads appeared darker blue, signifying higher histone content and lower DNA maturity. A total of 200 sperm were counted to assess and calculate the sperm DNA maturation rate using the formula: (number of mature sperm / total sperm) × 100%.

### Sperm acridine orange staining

A volume of 15 µL of sperm suspension was evenly spread on an anti-peeling slide and was allowed to air dry. The sample was fixed at room temperature for 15 min and then rinsed for 5 min. The slide was stained with 10 µg/mL acridine orange (AO) solution for 10 min in the dark. Under a fluorescence microscope, AO-bound sperm DNA emitted green fluorescence, while damaged DNA showed red fluorescence. A total of 200 sperm were counted to assess and calculate the DNA damage rate using the formula: (number of red fluorescence / number of green fluorescence) × 100%.

### Kit assays

The testicular tissue was weighed and an extraction solution from each kit was added at a ratio of 10:1 by mass. The mixture was homogenized at low temperature using a high-speed tissue grinder, then centrifuged at 500 × g for 15 min at 4 °C. The supernatant was collected. The kit instructions were followed precisely to measure the MDA content and SOD activity in the testicular tissue.

### ELISA

The reserved serum from Sect. 2.7 was utilized, and an ELISA kit was employed to quantify the levels of Testosterone, TNF-α, IL-6, and IL-1β in mouse serum. The impact of different SLBZS and febuxostat dose groups on these inflammatory markers was assessed.

### TUNEL analysis of testicular cells

After the paraffin sections of testicular tissue were dewaxed, the TUNEL staining kit instructions were followed and the slides were sealed with an anti-fluorescence quenching mounting solution. The staining results were examined immediately and images were captured using a fluorescence microscope. Testicular cells stained with DAPI exhibited blue fluorescence, while apoptotic cells displayed red fluorescence. The fluorescence was quantified using ImageJ. The cell apoptosis rate was calculated as: (number of red fluorescence / number of blue fluorescence) × 100%.

### Western blot analysis

Testicular tissue samples (50 mg) were homogenized in ice-cold immunoprecipitation lysis buffer with PMSF protease inhibitor using a high-speed tissue grinder. The homogenate was incubated on ice for 30 min, then centrifuged at 10,000 rpm for 15 min at 4 °C. The supernatant was collected, and protein concentration was measured by the BCA method. Proteins were prepared for polyacrylamide gel electrophoresis and transferred to a membrane via wet transfer. The membrane was blocked, washed, and incubated with the primary antibody overnight at 4 °C. After three washes, it was incubated with a 1:3,000-diluted HRP-conjugated goat anti-rabbit secondary antibody for 1 h at room temperature. Following three additional washes, the membrane was developed using enhanced chemiluminescence (ECL) solution. Grayscale analysis of the immunoblot was conducted using ImageJ software.

### Statistical analysis

Data analysis involved IBM SPSS Statistics 27 and GraphPad Prism 9.5. Measurement data were shown as mean ± SD, and categorical data as percentages. Data normality was assessed before analysis. Intergroup differences were analyzed by one-way ANOVA followed by Tukey’s HSD test; significance was defined as *p* < 0.05.

## Results

### SLBZS mass spectrometry and chromatography analysis

**Fig. 2:**
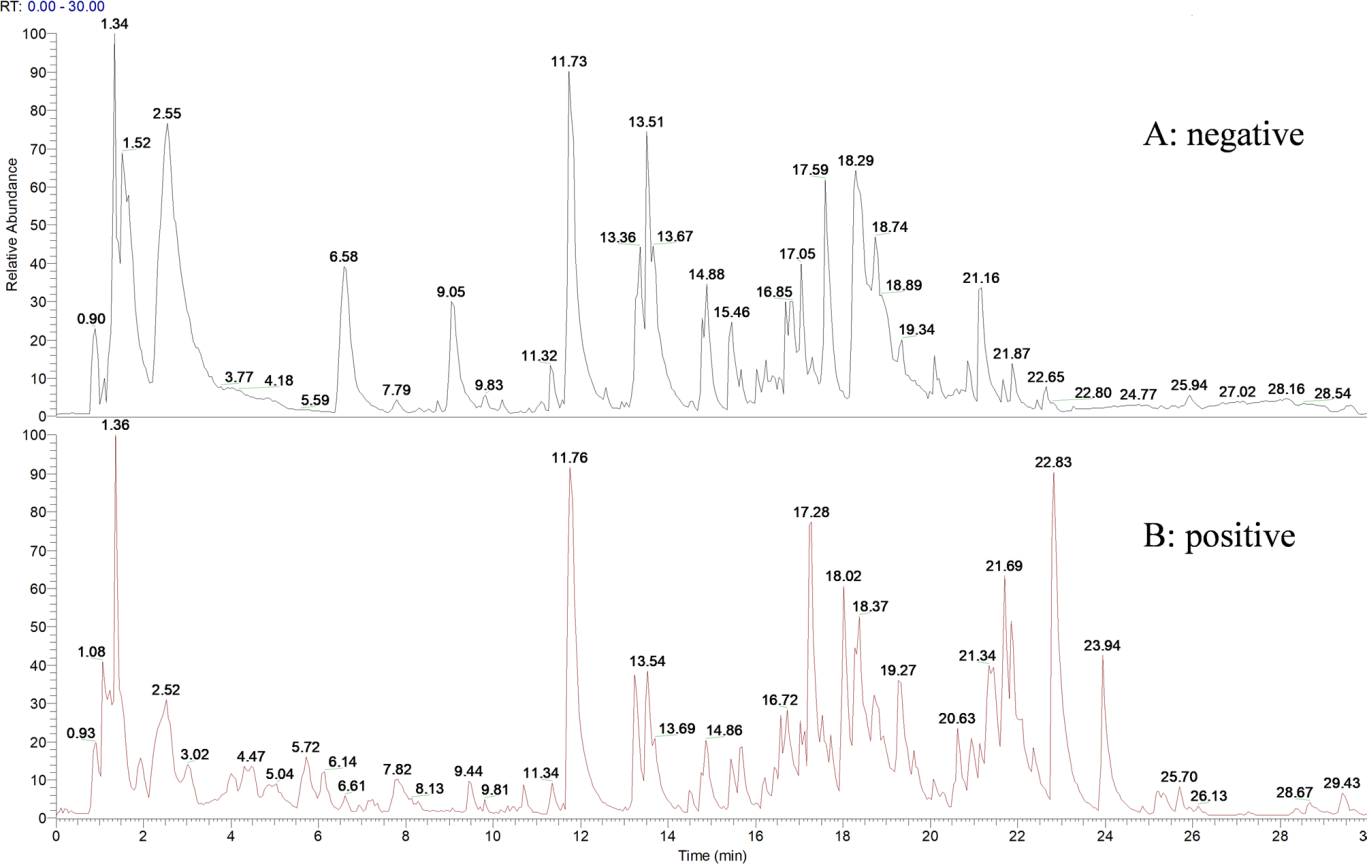
Total ion chromatogram of SLBZS. Negative ion mode (**A**). and Positive ion mode (**B**)

Chemical components of SLBZS were determined via Q-Orbitrap HR-LC-MS. Data analysis was performed using Compound Discoverer 3.3 (Thermo Fisher) and compared with the mzCloud database. Figure 2 shows the total ion chromatogram of SLBZS. Ultimately, 884 compounds were matched in mzCloud, with 260 compounds aligning completely with predicted components. Of these, 165 compounds achieved a comprehensive score above 60, including 145 in positive ion mode and 20 in negative ion mode (Table S1).

### Results of network Pharmacology

**Fig. 3:**
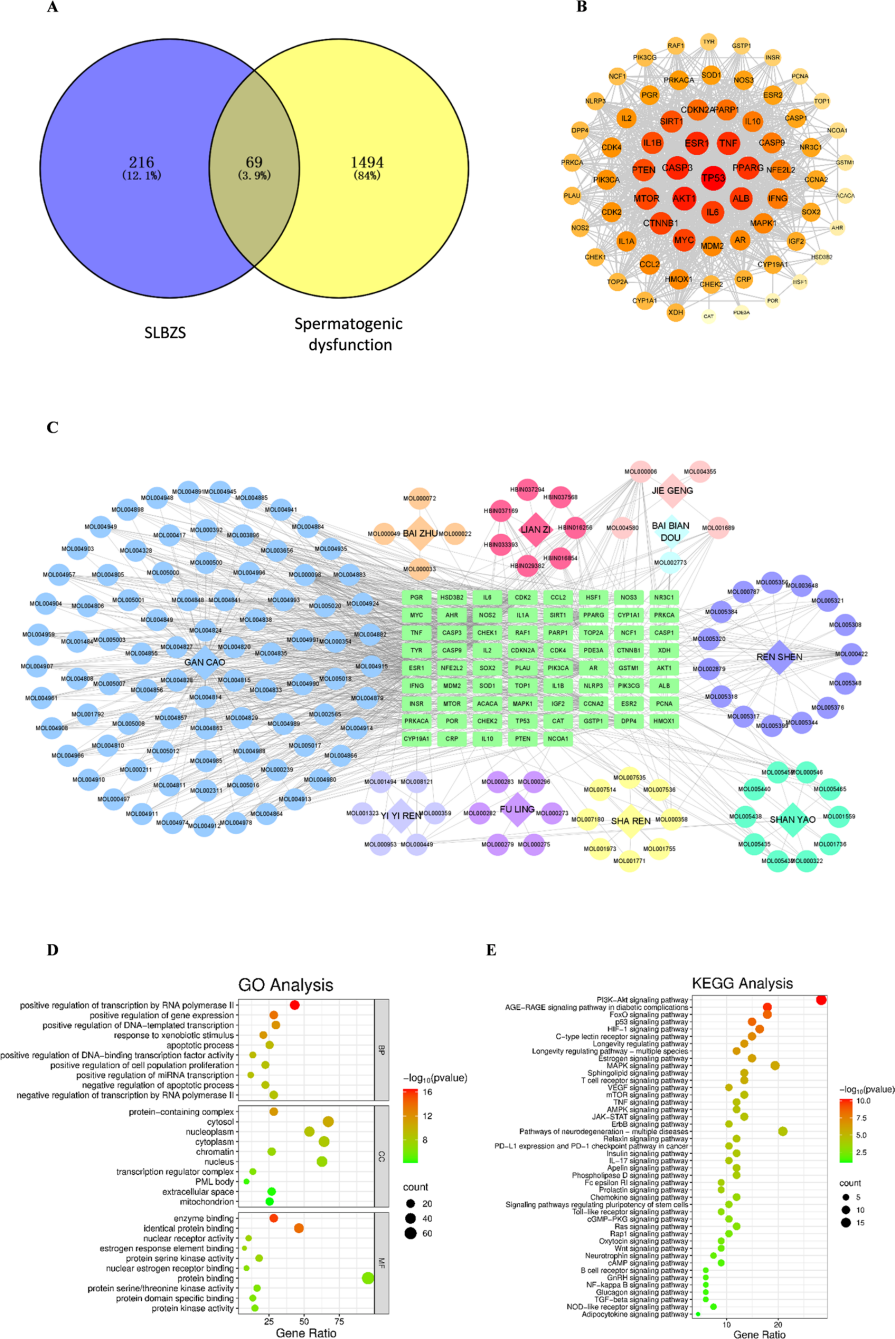
Results of network pharmacology. (**A**) Venn diagram. (**B**) PPI network diagram. (**C**) “Drug - ingredient - target - disease” network diagram. (**D**) GO functional enrichment analysis. (**E**) KEGG pathway enrichment analysis

Following retrieval, screening, and deduplication, 154 active components of SLBZS were identified, corresponding to 285 targets. Simultaneously, 1,563 disease targets associated with spermatogenic dysfunction were collected. By creating a Venn diagram, 69 common targets between the drug and the disease were pinpointed (Fig. 3(A)). Subsequently, a PPI analysis of these 69 targets was conducted using the STRING database. The resulting data were then imported into Cytoscape 3.8.2 to construct a PPI network highlighting targets closely linked to inflammation, such as IL-6, IL-1β, and TNF, which exhibited notably higher Degree values (Fig. 3(B)). Furthermore, a “drug-component-target-disease” network for treating spermatogenic dysfunction with SLBZS was established using Cytoscape 3.8.2 (Fig. 3(C)). The intersection genes between the drug and the disease were selected for G functional enrichment analysis through the DAVID database (Fig. 3(D)). Additionally, a KEGG pathway enrichment analysis was performed. Signaling pathways with a *P* value below 0.05 were identified, and a bubble chart was generated based on the number of genes enriched in each pathway. The bubble size represented the gene count, while the color intensity indicated the level of significance (Fig. 3(E)). Enriched pathways identified through KEGG pathway analysis included the MAPK signaling pathway, TNF signaling pathway, and NF-κB signaling pathway, in addition to apoptosis-related pathways. These results indicate that SLBZS may improve spermatogenic dysfunction by influencing inflammatory response and cell apoptosis mechanisms.

### Hyperuricemia affects spermatogenic function in mice

**Fig. 4:**
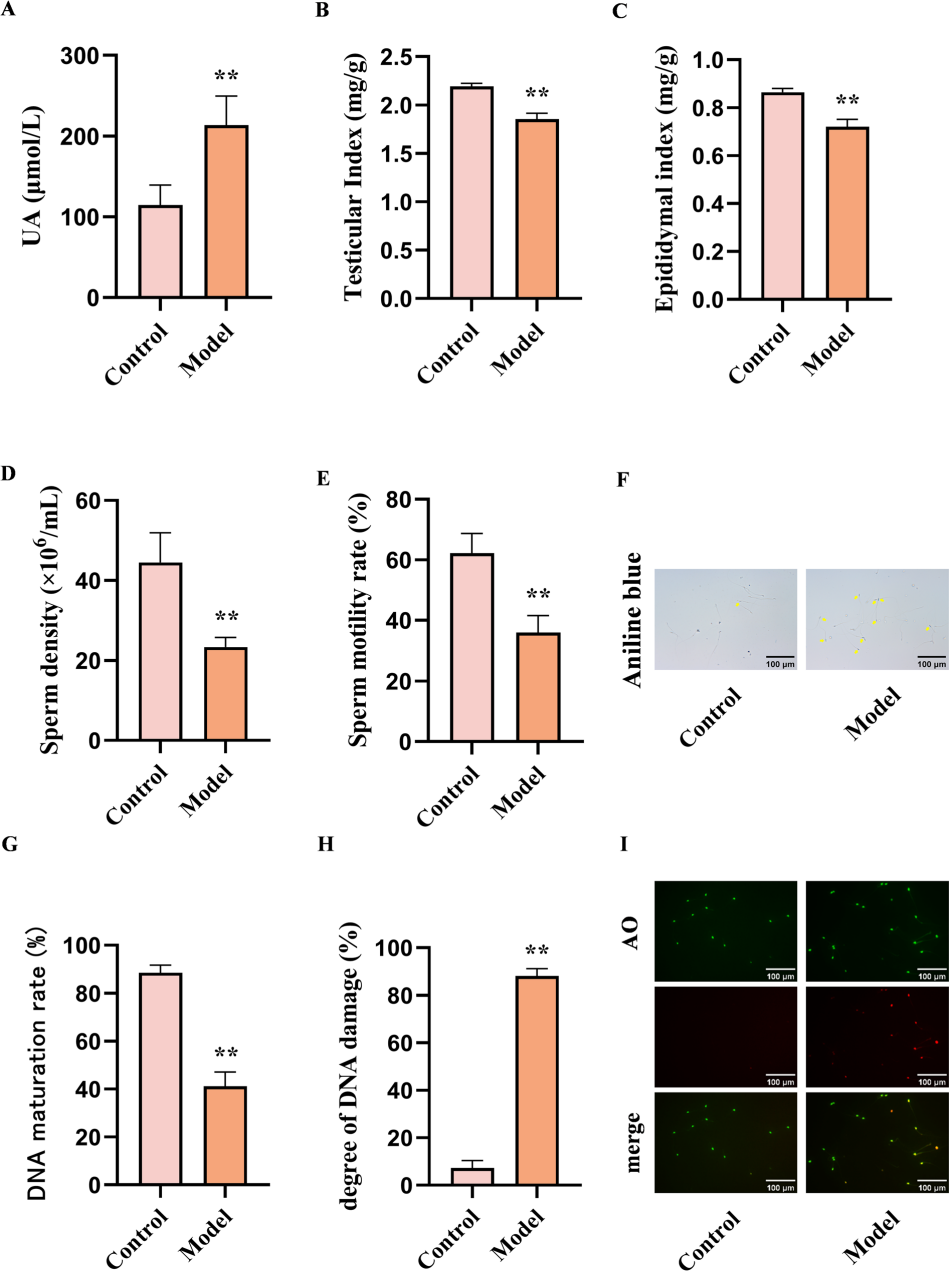
Hyperuricemia leads to spermatogenic dysfunction in mice. (**A**) Serum uric acid content in mice. (**B**) Testis index. (**C**) Epididymis index. (**D**) Sperm density. (**E**) Sperm motility rate. (**F**) Sperm aniline blue staining. The yellow arrow indicates DNA immature sperm. (**G**) Sperm DNA maturation rate. (**H**) Degree of sperm DNA damage. (**I**) Sperm AO staining. Red fluorescence indicates sperm with DNA damage. Data are shown as the means ± SEM. ^**^p < 0.01. Statistical comparisons between two groups were performed using Student’s t-test

The initial experiment demonstrated a significant increase in serum uric acid levels in the model group mice (*p* < 0.01) (Fig. 4(A)), alongside a notable reduction in both testicular and epididymal indices compared to the control group (*p* < 0.01) (Fig. 4(B, C)). An automatic sperm analyzer detected a significant decline in sperm density and the percentage of progressively motile sperm in the model group (*p* < 0.01) (Fig. 4(D, E)). Sperm aniline blue staining and acridine orange staining revealed decreased maturity of sperm nuclear proteins and elevated DNA damage in the model group (*p* < 0.01) ((Fig. 4F-I)). These findings collectively confirm that hyperuricemia impairs spermatogenic function in mice, validating the model’s successful establishment.

### SLBZS improves testicular index, epididymal index and uric acid level in hyperuricemic mice

In the model group, mice exhibited significant reductions in body weight, testicular index, and epididymal index (*p* < 0.01), as depicted in Fig. 1(B–E). The high-dose (20.14 g/kg) SLBZS group notably improved these parameters (*p* < 0.01 or *p* < 0.05). While the medium-dose groups (10.07 g/kg) and low-dose groups (5.04 g/kg) showed a trend towards improvement, the changes were not statistically significant. The *P* values for the low-dose group are 0.6269, 0.9930, and 0.7052, respectively, while those for the medium-dose group are 0.2983, 0.3812, and 0.2098, respectively. Additionally, the model group demonstrated a marked increase in serum uric acid levels (*p* < 0.01), which SLBZS treatment (10.07 g/kg and 20.14 g/kg) effectively reduced (*p* < 0.01 and *p* < 0.05), as shown in Fig. 1(F).

### SLBZS ameliorates the histopathological lesions of testicular tissue in hyperuricemia mice

In the H&E-stained section of testicular tissue (Fig. 1(G)), the control group exhibits densely packed seminiferous tubules with thin membranes, oval or circular lumens, and intact structures. Supporting and spermatogenic cells at various developmental stages are orderly, with mature sperm visible in the lumen. In contrast, the model group shows loosely distributed, atrophic tubules with thicker membranes. Immature spermatogenic cells are detached, and sperm count in the lumen is reduced. Treatment with SLBZS or febuxostat ameliorates these lesions to varying extents.

In the model group, the seminiferous tubules’ inner diameter and germ cell layer score decreased, while the peritubular membrane thickness increased, compared to the control group (*p* < 0.01). Treatment with SLBZS led to an increase in the seminiferous tubules’ inner diameter, although the high-dose group (20.14 g/kg) showed no statistically significant change, an upward trend was observed (*p* = 0.0809) (Fig. 1(H)). Post-SLBZS treatment, the peritubular membrane thickness decreased, and the germ cell layer score increased. The high-dose SLBZS group (20.14 g/kg) showed lower seminiferous tubule basement membrane thickness (2.46 ± 0.31, *P* < 0.01 vs. model group; febuxostat: 2.55 ± 0.30) and higher spermatogenic cell layer scores (3.43 ± 0.31, *P* < 0.01 vs. model group; febuxostat: 3.32 ± 0.28), indicating better outcomes compared to the febuxostat group. (Fig. 1(I, J)).

### SLBZS improves sperm quality in hyperuricemic mice

**Fig. 5:**
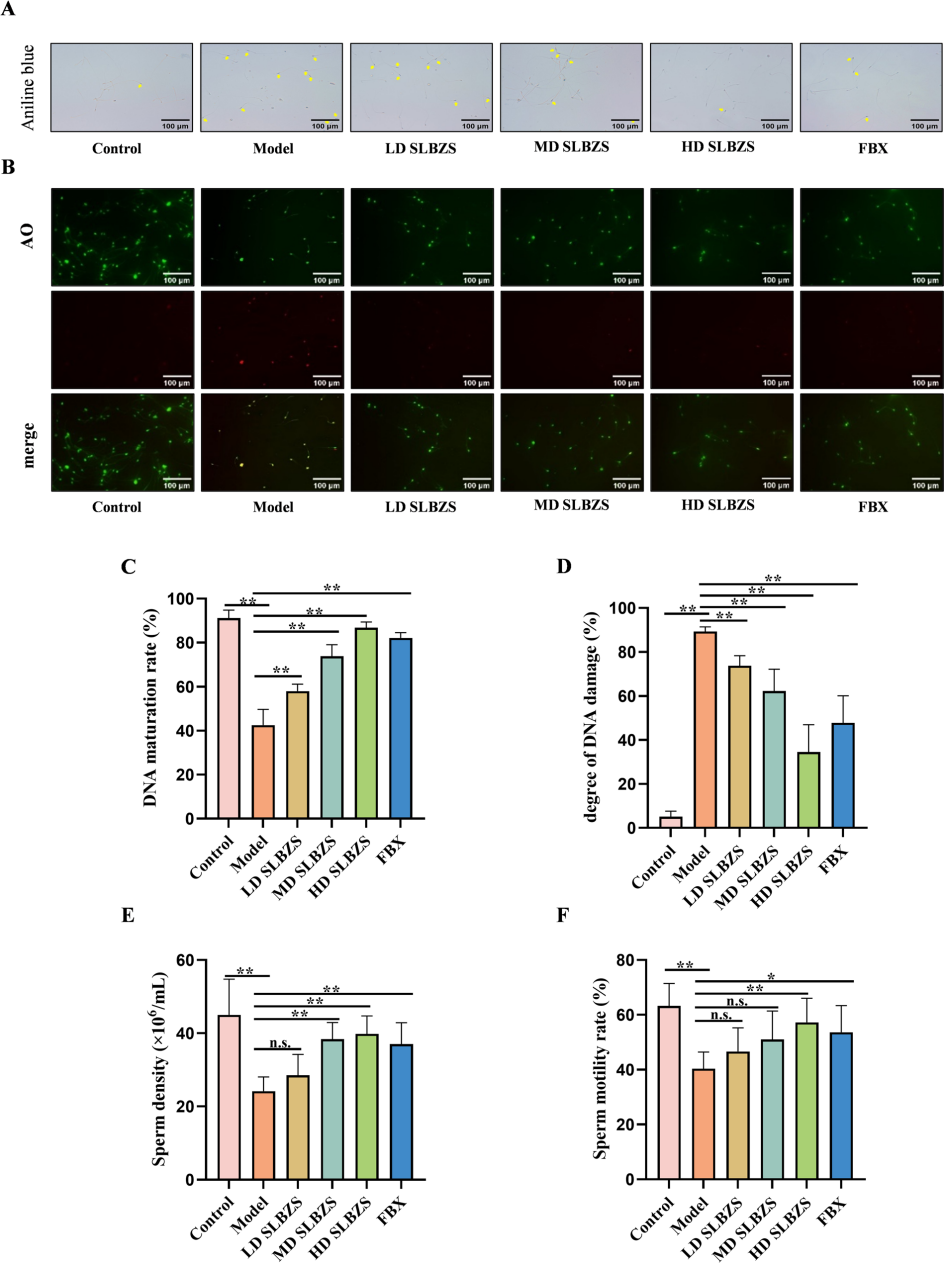
SLBZS improves the sperm quality of mice. (**A**) Sperm aniline blue staining. The yellow arrow indicates DNA immature sperm. (**B**) Sperm AO staining. Red fluorescence indicates sperm with DNA damage. (**C**) Sperm DNA maturation rate. (**D**) Degree of sperm DNA damage. (**E**) Sperm density. (**F**) Sperm motility rate. Data are shown as the means ± SEM. ^*^p < 0.05; ^**^p < 0.01

Aniline blue staining reveals that immature sperm heads are stained blue, while mature sperm heads remain unstained or faintly stained (Fig. 5(A)). Figure 5(C) demonstrates a marked reduction in sperm DNA maturity in the model group. SLBZS treatment enhances sperm DNA maturity, with the high-dose group (20.14 g/kg) showing the most pronounced improvement (*p* < 0.01). Figure 5(B, D) indicates a significant increase in red fluorescence in sperm AO staining in the model group, suggesting exacerbated DNA damage. SLBZS treatment mitigates this damage (*p* < 0.01). An automatic sperm analyzer confirms a significant decline in sperm density and motility in the model group. The high-dose SLBZS group (20.14 g/kg) significantly enhances sperm quality (*p* < 0.01). (Fig. 5(E, F)).

### SLBZS improves oxidative stress and inflammatory indexes in hyperuricemic mice

**Fig. 6:**
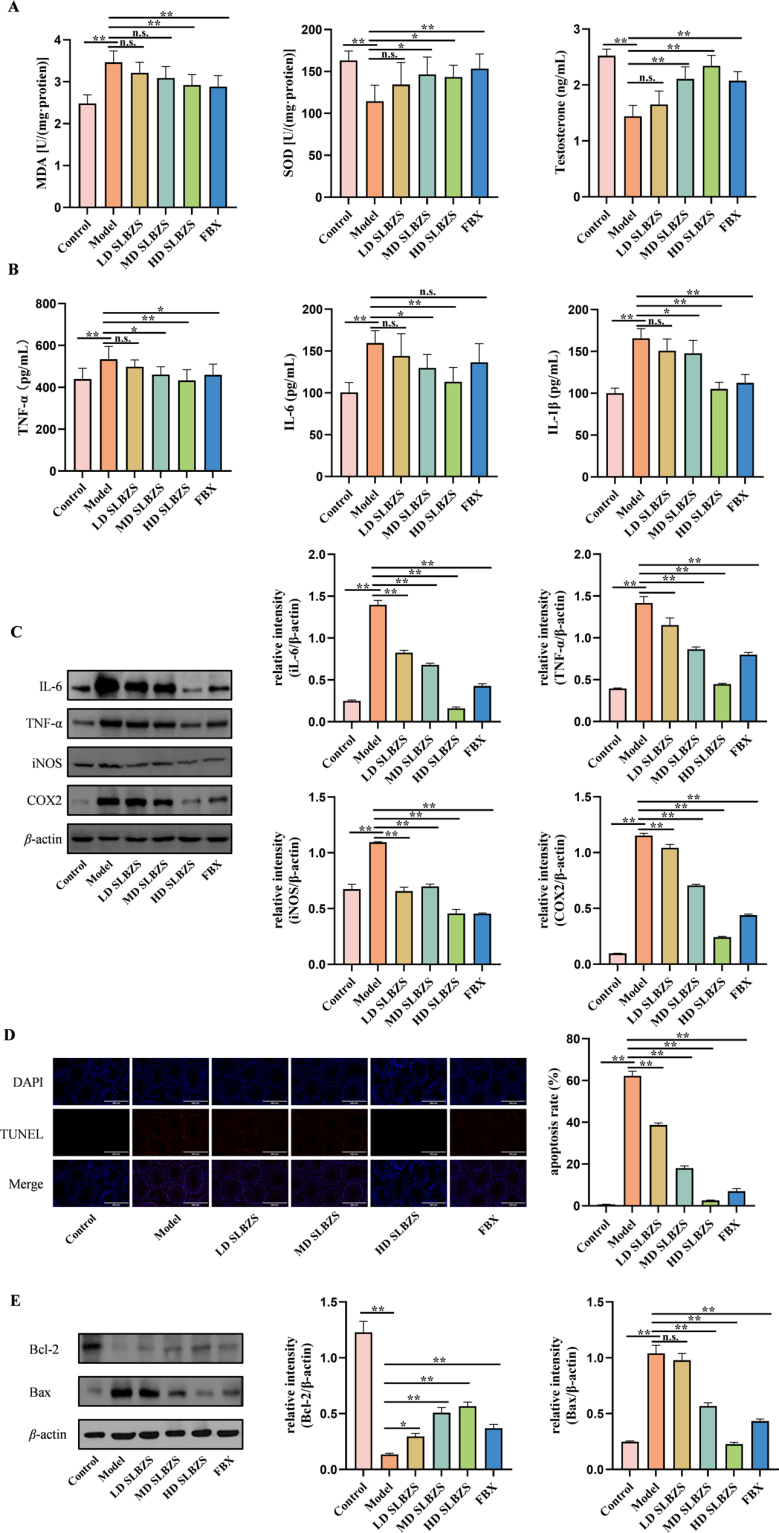
SLBZS improved testicular oxidative stress in HUA mice, alleviated serum inflammatory indicators, and reduced testicular cell apoptosis. (**A**) MDA content, SOD activity and testosterone levels. (**B**) Serum inflammatory indexes TNF-α, IL-6, and IL-1β. (**C**) The expression levels of IL-6, TNF-α, iNOS and COX2 proteins were detected by Western blot. (**D**) TUNEL staining of testicular tissue and testicular cell apoptosis rate. (**E**) The expression levels of Bcl-2 and Bax proteins were detected by Western blot. Data are shown as the means ± SEM. ^*^p < 0.05; ^**^p < 0.01

The MDA content, SOD activity and testosterone levels in testicular tissue were assessed (Fig. 6(A)). In the model group, MDA levels increased while SOD activity and testosterone levels decreased compared to the control (*p* < 0.01). High-dose SLBZS (20.14 g/kg) significantly reduced MDA content (*p* < 0.01), with a decreasing trend observed in low-dose (5.04 g/kg) (*P* = 0.3710) and medium-dose groups (10.07 g/kg) (*P* = 0.0508), though not statistically significant. SOD activity and testosterone levels increased in both medium-dose (10.07 g/kg) and high-dose SLBZS groups (20.14 g/kg) relative to the model group (*p* < 0.05 or *P* < 0.01). Serum inflammatory markers were measured using ELISA (Fig. 6(B)), revealing significant elevations in TNF-α, IL-6, and IL-1β in the model group (*p* < 0.01). Treatment with medium (10.07 g/kg) and high doses (20.14 g/kg) of SLBZS significantly decreased these markers (*p* < 0.05 or *p* < 0.01), with the high-dose group showing the most pronounced effect (*p* < 0.01). The low-dose group exhibited a decreasing trend without statistical significance (*P* = 0.6673, 0.5666, 0.1069). Western blot analysis of testicular tissues showed increased IL-6, TNF-α, iNOS and COX-2 protein expressions in the model group, which were effectively inhibited by SLBZS treatment (*p* < 0.01) (Fig. 6(C)).

### SLBZS improves testicular cell apoptosis

To assess the impact of SLBZS on testicular cell apoptosis, TUNEL staining was employed. Figure 6(D) illustrates that red fluorescence indicates apoptotic cells. Apoptosis was markedly elevated in the model group (*p* < 0.01), while SLBZS treatment ameliorated this condition (*p* < 0.01). Additionally, Western blot analysis was conducted to examine Bcl-2 and Bax protein levels. The model group exhibited a significant decrease in Bcl-2 and an increase in Bax expression (*p* < 0.01). SLBZS treatment upregulated Bcl-2 and inhibited Bax expression (*p* < 0.01), with the high-dose group showing superior effects compared to the febuxostat group (Fig. 6(E)).

### SLBZS inhibits the MAPK/NF-κB signaling pathway

**Fig. 7:**
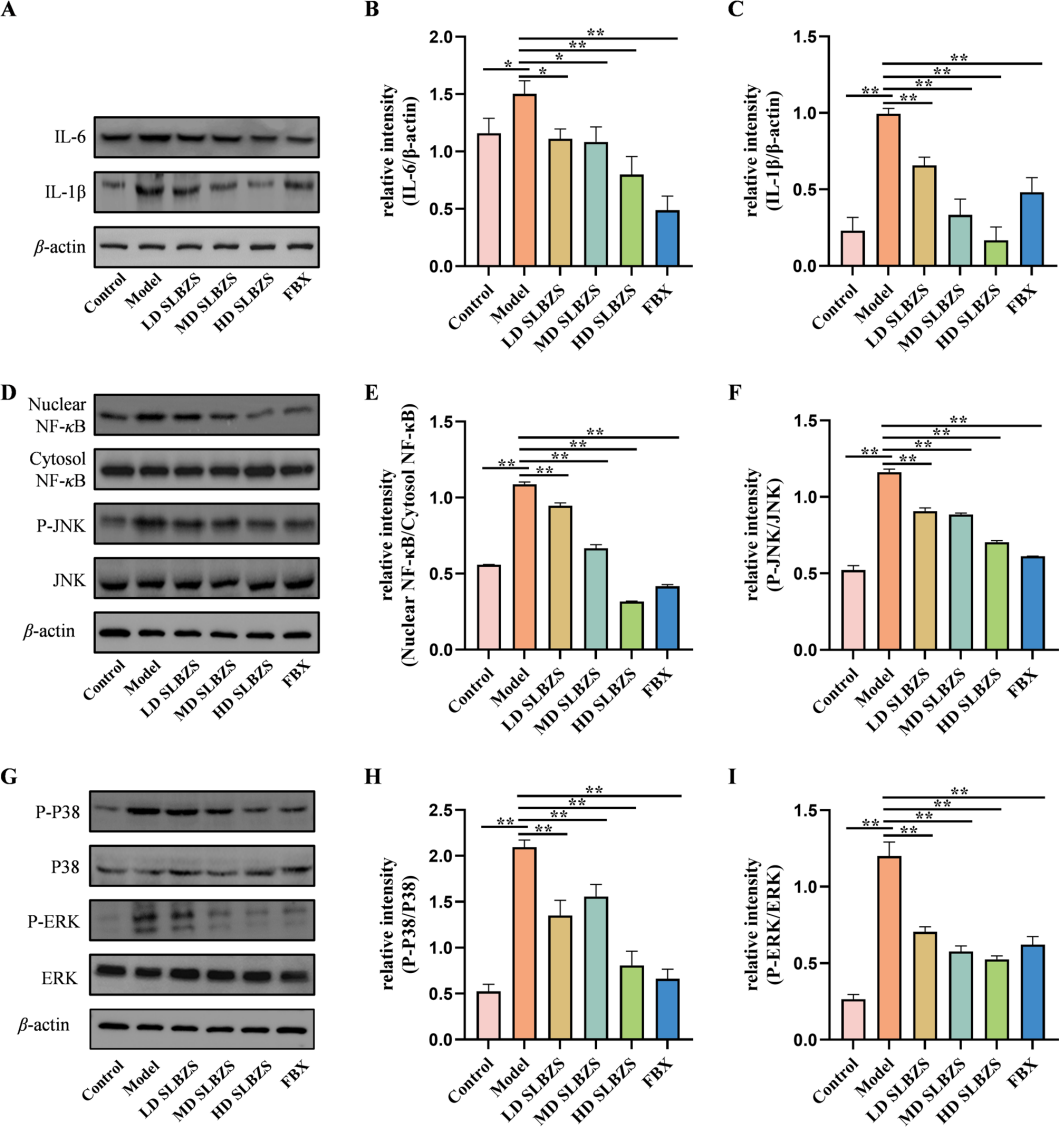
SLBZS treatment inhibits the MAPK/NF-κB signaling pathway. (**A-I**) The protein expression levels of IL-6, IL-1β, NF-кB, and MAPKs were detected by Western blot and gray value analysis was performed. Data are shown as the means ± SEM. ^*^p < 0.05; ^**^p < 0.01

We employed Western blot analysis to examine proteins associated with the MAPK/NF-κB signaling pathway. Figure 7(A–I) illustrates a marked increase in IL-6, IL-1β expression, and NF-κB nuclear translocation in the model group (*p* < 0.05 or *p* < 0.01). SLBZS treatment significantly suppressed IL-6, IL-1β expression, and NF-κB nuclear translocation (*p* < 0.05 or *p* < 0.01). Additionally, the model group showed elevated MAPK phosphorylation (*p* < 0.01), which was reduced by SLBZS treatment (*p* < 0.01).

### 10. SLBZS inhibits the NLRP3 inflammasome pathways

**Fig. 8:**
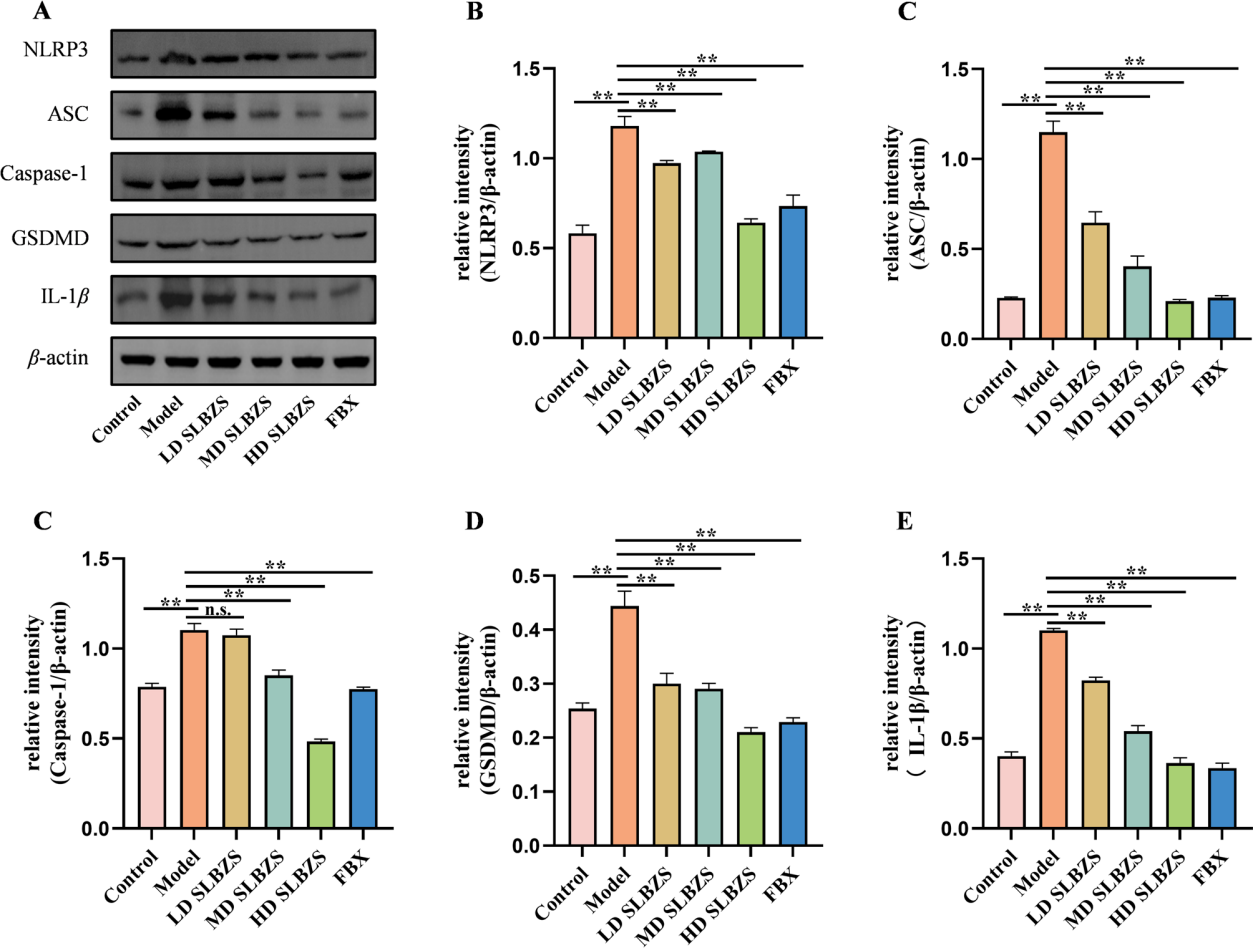
SLBZS inhibits the NLRP3 inflammasome pathways. (**A-E**) The protein expression levels of NLRP3, ASC, caspase-1, GSDMD, and IL-1β were detected by Western blot and and gray value analysis was performed. Data are shown as the means ± SEM. ^*^p < 0.05; ^**^p < 0.01

Similarly, we also employed Western blot analysis to examine proteins associated with the NLRP3 inflammasome pathways (Fig. 8). The model group exhibits significantly elevated levels of NLRP3, ASC, caspase-1, GSDMD, and IL-1β compared to the control (*p* < 0.01). SLBZS treatment reduces the expression of these proteins across various doses, with the high-dose group (20.14 g/kg) demonstrating the most pronounced inhibition of NLRP3, ASC, caspase-1, and GSDMD (*p* < 0.01).

## Discussion

The incidence of male spermatogenic dysfunction is rising, exacerbating infertility and imposing significant economic, psychological, and social burdens on patients and healthcare systems [21]. Current treatment strategies remain limited. Thus, investigating the causes of testicular spermatogenic dysfunction and enhancing spermatogenic function at its source is crucial.

Recent studies have identified HUA as a significant contributor to testicular spermatogenic dysfunction. Clinical data indicate a negative correlation between serum UA levels and sperm quality in male HUA patients; higher UA levels are associated with poorer sperm quality [22]. Our previous research corroborates these findings, showing a marked decline in sperm quality in HUA mice. Additionally, these mice exhibited decreased SOD and catalase (CAT) activity in testicular tissue, alongside increased MDA levels, oxidative damage, and apoptosis [23]. Thus, effectively lowering serum UA can mitigate testicular damage and spermatogenic dysfunction associated with elevated UA.

Research on the reduction of uric acid using traditional Chinese medicine (TCM) has a history spanning thousands of years. As a crucial component of complementary and alternative medicine, TCM is pivotal in uric acid reduction treatments and is utilized globally. SLBZS, a TCM compound comprising herbs like ginseng and Atractylodes macrocephala, is known for tonifying qi, strengthening the spleen, draining dampness, and halting diarrhea. Clinically, it has been demonstrated to lower serum uric acid, regulate serum IL-6, TNF-α, and erythrocyte sedimentation rate [24, 25], and alleviate arthritis induced by HUA [26]. Animal studies further confirm that intragastric administration of SLBZS significantly reduces uric acid levels in HUA model mice.

In this study, the chemical constituents of SLBZS were identified using Q-Orbitrap high-resolution liquid chromatography-mass spectrometry. Through precise matching with predicted components, mzCloud retrieval, and a comprehensive score exceeding 60, we identified 145 compounds in positive ion mode and 20 in negative ion mode, underscoring the complexity of SLBZS’s chemical composition. The specific properties and interactions of these components remain unknown. Among the compounds obtained from SLBZS, it is reported that ginsenosides can directly react with free radicals and remove them, thereby reducing oxidative damage to testicular tissue by free radicals [27]; atractylenolide can inhibit the release of inflammatory mediators such as TNF-α, IL-6, and IL-1β, reducing inflammation [28]; atractylodes oil prevents NF-κB activation, hindering its nuclear entry and the transcription of inflammation-related genes, thereby exerting anti-inflammatory effects [29]. Additionally, SLBZS contains various triterpenoids and saponins that regulate cell metabolism, proliferation, and differentiation, contributing to spermatogenesis and sperm maturation.

Employing network pharmacology, we conducted a comprehensive analysis to elucidate the relationship between SLBZS and spermatogenic dysfunction. Systematic screening identified 69 overlapping targets. Constructing a PPI network and performing GO and KEGG enrichment analyses revealed that SLBZS likely ameliorates spermatogenic dysfunction through a multi-target, multi-pathway mechanism. The identified pathways are implicated in the regulation of inflammatory responses and cell apoptosis processes, which are crucial in the pathophysiology of spermatogenic dysfunction.

In this study, we first established a hyperuricemia-induced model of spermatogenic dysfunction. Our findings demonstrated that SLBZS: 1 significantly enhanced testicular and epididymal indexes, sperm density, and motility in HUA model mice, improved sperm DNA maturity, and reduced DNA damage; 2 alleviated histopathological lesions in testicular tissue, reversing indicators of spermatogenic function such as seminiferous tubule diameter, peritubular membrane thickness, and germ cell layer count; 3 decreased oxidative stress levels in testicular tissue.

Research indicates that HUA disrupts the cellular oxidative-antioxidant balance, leading to oxidative stress and triggering inflammation in the testicular seminiferous epithelium. Inflammation adversely affects spermatogenesis by targeting cell junction proteins, compromising the integrity of the blood-testis barrier [30, 31]. The blood-testis barrier is crucial for the stability of the microenvironment in the testis. Once it is damaged, a series of chain reactions will be triggered [32]. Moreover, there will be excessive secretion of inflammatory mediators. This excessive secretion will inhibit phagocytic function and then induce the occurrence of inflammatory innate immune responses [33]. This immune response can induce apoptosis [34, 35], progressively damaging spermatogenic and Sertoli cells in the testicular seminiferous tubules. Over time, this results in the complete loss of seminiferous epithelial function, significantly impairing male spermatogenic capacity. Whether Songling and Kidney-tonifying herbs enhance spermatogenic function in hyperuricemia mice by alleviating oxidative stress, inhibiting inflammation, and reducing apoptosis remains to be confirmed.

**Fig. 9:**
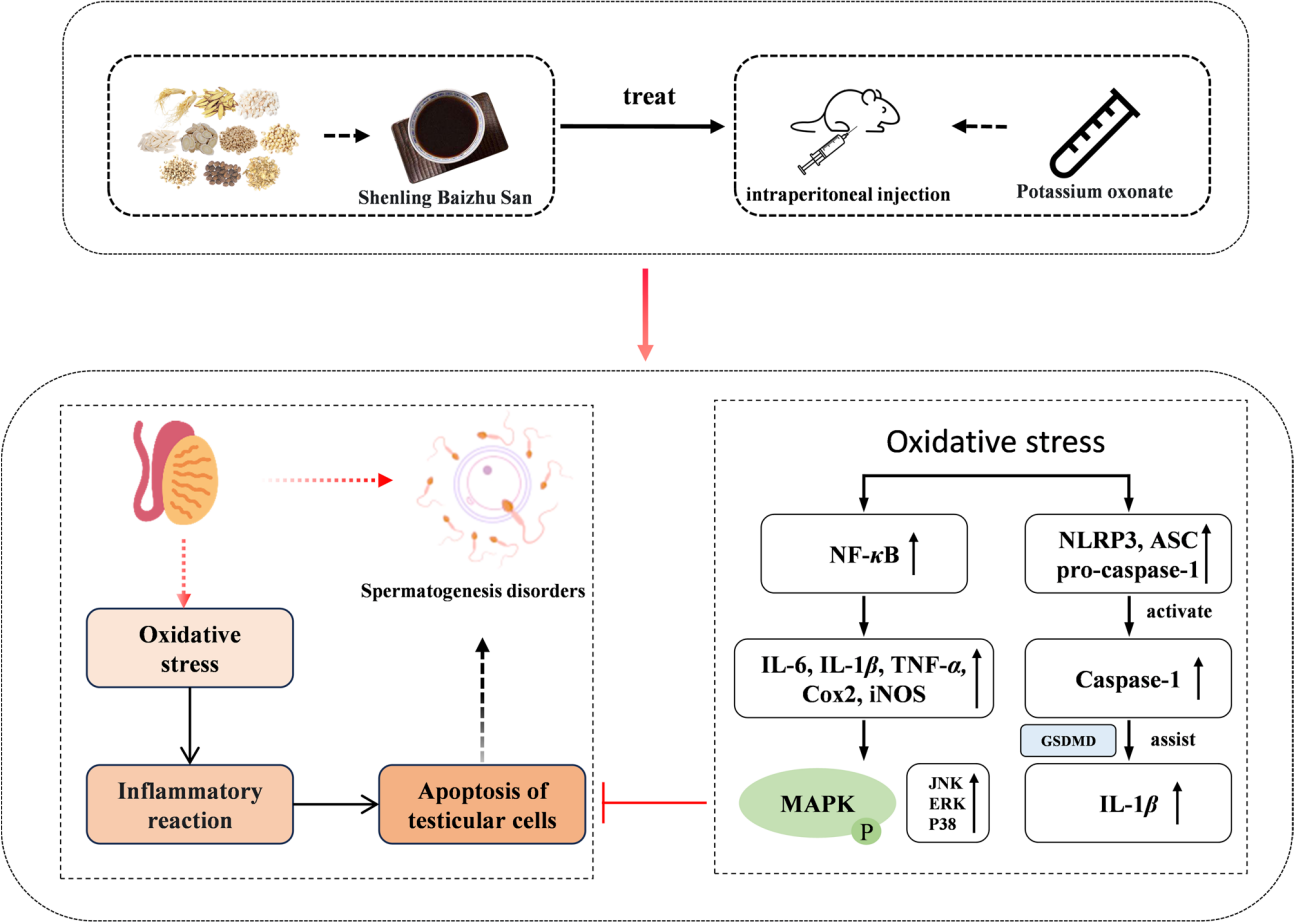
Mechanism schematic diagram

To investigate the mechanism by which SLBZS enhances spermatogenesis in HUA mice, we examined the involvement of inflammatory signaling pathways. NF-κB is a pivotal regulator of pro-inflammatory and anti-inflammatory mediators [36]. Activation of NF-κB upregulates IL-6 and IL-1β expression [37]. MAPKs, such as JNK, P38, and ERK, are crucial in modulating cellular responses to cytokines [38]. Phosphorylation of MAPKs is linked to IL-6 and IL-1β activation [39, 40]. The NLRP3 inflammasome, a large cellular multiprotein complex, is vital in innate immunity, comprising NLRP3, apoptosis-associated speck-like protein containing a caspase recruitment domain (ASC) with pro-caspase-1 [41]. It recognizes pathogen-associated molecular patterns (PAMPs) and damage-associated molecular patterns (DAMPs), activating caspase-1. In the presence of NF-κB, it processes IL-1β and IL-18 precursors into mature forms [42, 43]. GSDMD facilitates cell membrane perforation, aiding in the release of IL-1β and IL-18 [44], suppressing phagocytosis, and inducing an inflammatory response, finely regulating the immune process. Western blot analysis revealed that SLBZS reduced P38, JNK, and ERK phosphorylation, inhibited NF-κB nuclear translocation, and decreased the expression of iNOS, COX-2, IL-6, NLRP3, caspase-1, GSDMD, and IL-1β. TUNEL staining assessed testicular cell apoptosis rates, while Western blot analysis of Bcl-2 and Bax examined apoptosis-related proteins. SLBZS upregulated Bcl-2 and downregulated Bax, effectively suppressing testicular cell apoptosis. These findings suggest that SLBZS may enhance testicular spermatogenesis in HUA mice by mitigating inflammation through the MAPK/NF-κB and NLRP3 inflammasome pathways, reducing oxidative stress, and inhibiting testicular cell apoptosis. Refer to Fig. 9 for a schematic representation.

Reduction of apoptosis and inflammation indicates that SLBZS may be superior to febuxostat in preventing chronic testicular injury, but the issue of its bioavailability requires further formulation optimization. Moreover, the specific active compounds responsible for these effects remain to be elucidated, and future studies will focus on their isolation and mechanistic validation.

## Conclusions

This study showed that SLBZS reduces UA levels and inhibits the MAPK/NF-κB and NLRP3 inflammasome pathways, thereby decreasing local inflammation, preventing testicular cell apoptosis, and enhancing spermatogenic function in HUA mice, offering a foundation for clinical application. However, despite its role in strengthening the spleen and eliminating dampness, SLBZS has limited clinical experience in treating male infertility, necessitating further investigation into its efficacy.

## Supplementary Information

Below is the link to the electronic supplementary material.

Supplementary Material 1

Supplementary Material 2

## Data Availability

The datasets used and/or analysed during the current study are available from the corresponding author on reasonable request.
