# Supplementary material for: Shenling Baizhu San attenuates testicular spermatogenic dysfunction in hyperuricemic mice via dual modulation of MAPK/NF-κB and NLRP3 inflammasome pathways
Source: Hereditas. 2025 Sep 29;162:195. doi: 10.1186/s41065-025-00553-x (PMC12482675; doi:10.1186/s41065-025-00553-x)
Supplement: Supplementary file 1 — Supplementary Material 1 [file 41065_2025_553_MOESM1_ESM.docx]

Table S1. The main compounds of SLBZS and their mass spectrometry data.

| No. | Compounds | Formula | Library  score | RT  (min) | m/z | Area |
| --- | --- | --- | --- | --- | --- | --- |
| 1 | Adenosine | C10 H13 N5 O4 | 100 | 4.321 | 268.10379 | 6318615311 |
| 2 | Ononin | C22 H22 O9 | 99.9 | 13.259 | 431.13278 | 7502451784 |
| 3 | Formononetin | C16 H12 O4 | 99.9 | 15.679 | 269.08032 | 4359411422 |
| 4 | Isoliquiritigenin | C15 H12 O4 | 99.8 | 11.772 | 257.07965 | 24980109602 |
| 5 | 2,3,4,9-Tetrahydro-1H-β-carboline-3-carboxylic acid | C12 H12 N2 O2 | 99.7 | 8.426 | 217.09708 | 365824498.3 |
| 6 | Docosanamide | C22 H45 N O | 99.6 | 25.357 | 340.35681 | 1714097932 |
| 7 | α-Eleostearic acid | C18 H30 O2 | 99.6 | 19.231 | 279.23138 | 6526131012 |
| 8 | Palmitoyl ethanolamide | C18 H37 N O2 | 99.5 | 21.241 | 300.28925 | 408191419.4 |
| 9 | Di(2-ethylhexyl) phthalate | C24 H38 O4 | 99.4 | 22.791 | 391.28333 | 523092225.7 |
| 10 | Linoleoyl ethanolamide | C20 H37 N O2 | 99.4 | 20.792 | 324.28912 | 932800677.1 |
| 11 | Citroflex A-4 | C20 H34 O8 | 99.4 | 19.245 | 403.23193 | 849083764 |
| 12 | Nicotinic acid | C6 H5 N O2 | 99.4 | 2.018 | 124.0395 | 764323699.2 |
| 13 | (-)-Camphor | C10 H16 O | 99.4 | 15.34 | 153.12727 | 626819321.5 |
| 14 | Oleoyl ethanolamide | C20 H39 N O2 | 99.3 | 21.555 | 326.30493 | 227951474 |
| 15 | Trigonelline | C7 H7 N O2 | 99.2 | 1.422 | 138.05478 | 11351240707 |
| 16 | Stearamide | C18 H37 N O | 99.2 | 22.531 | 284.29449 | 846262420.6 |
| 17 | 5-Hydroxymethyl-2-furaldehyde | C6 H6 O3 | 99.1 | 5.729 | 127.03911 | 8197267586 |
| 18 | Nicotinamide | C6 H6 N2 O | 99.1 | 2.099 | 123.05553 | 1194355876 |
| 19 | L(-)-Pipecolinic acid | C6 H11 N O2 | 99.1 | 1.554 | 130.08629 | 6482051943 |
| 20 | Monoolein | C21 H40 O4 | 99 | 21.945 | 379.28122 | 1572452524 |
| 21 | 1-Linoleoyl glycerol | C21 H38 O4 | 98.9 | 21.217 | 377.26538 | 3630203254 |
| 22 | 18-β-Glycyrrhetinic acid | C30 H46 O4 | 98.8 | 18.312 | 453.33517 | 13753436285 |
| 23 | 3-Hydroxypicolinic acid | C6 H5 N O3 | 98.8 | 9.478 | 140.03426 | 223308650.6 |
| 24 | Isokaempferide | C16 H12 O6 | 98.8 | 15.908 | 301.0704 | 358659131.9 |
| 25 | 18-β-Glycyrrhetinic acid | C30 H46 O4 | 98.8 | 20.635 | 471.3461 | 5568955145 |
| 26 | Scoparone | C11 H10 O4 | 98.8 | 12.318 | 207.06519 | 240783931 |
| 27 | Norharman | C11 H8 N2 | 98.7 | 8.702 | 169.07622 | 173924468.2 |
| 28 | 9-Oxo-10(E),12(E)-octadecadienoic acid | C18 H30 O3 | 98.6 | 19.274 | 295.22638 | 2624648578 |
| 29 | 7-hydroxy-6-methoxy-2H-chromen-2-one | C10 H8 O4 | 98.3 | 11.335 | 193.0495 | 659580636.8 |
| 30 | Adenine | C5 H5 N5 | 98.3 | 4.321 | 136.06181 | 1099946169 |
| 31 | Methyl picolinate | C7 H7 N O2 | 98.2 | 9.328 | 138.05519 | 263298564.3 |
| 32 | (2Z)-6-hydroxy-2-[(4-hydroxy-3-methoxyphenyl)methylidene]-2,3- dihydro-1-benzofuran-3-one | C16 H12 O5 | 98.2 | 14.247 | 285.07584 | 759748776.1 |
| 33 | 2-methyl-1,2-dihydrophthalazin-1-one | C9 H8 N2 O | 98.2 | 11.466 | 161.07095 | 90084348.35 |
| 34 | Choline | C5 H13 N O | 98.1 | 1.285 | 104.10725 | 3440491740 |
| 35 | Choline | C5 H13 N O | 98 | 1.174 | 104.10735 | 3151235976 |
| 36 | 5'-S-Methyl-5'-thioadenosine | C11 H15 N5 O3 S | 97.9 | 7.835 | 298.09695 | 132650140.6 |
| 37 | Naringeninchalcone | C15 H12 O5 | 97.6 | 11.517 | 273.07562 | 185721653.8 |
| 38 | Naringeninchalcone | C15 H12 O5 | 97.6 | 11.772 | 273.07504 | 339587050.1 |
| 39 | 4-[(3R)-5,7-dimethoxy-6-(3-methylbut-2-en-1-yl)-3,4-dihydro-2H-1- benzopyran-3-yl]-2-(3-methylbut-2-en-1-yl)benzene-1,3-diol | C27 H34 O5 | 97.6 | 19.385 | 439.24719 | 2564806974 |
| 40 | Naringeninchalcone | C15 H12 O5 | 97.6 | 12.559 | 273.07559 | 685340681.9 |
| 41 | 4-(8-Isopropenyl-3,4,8,9-tetrahydro-2H-furo[2,3-H]chromen-3-yl)-1,3- benzenediol | C20 H20 O4 | 97.5 | 17.421 | 325.14282 | 1546968660 |
| 42 | 3-(propan-2-yl)-octahydropyrrolo[1,2-a]pyrazine-1,4-dione | C10 H16 N2 O2 | 97.2 | 9.1 | 197.1286 | 143191255.6 |
| 43 | Hexadecanamide | C16 H33 N O | 96.9 | 21.344 | 256.26306 | 502470506.8 |
| 44 | Glycitein | C16 H12 O5 | 96.8 | 14.798 | 285.07526 | 491232530.5 |
| 45 | D-(+)-Pyroglutamic Acid | C5 H7 N O3 | 96.7 | 2.517 | 130.04988 | 16614609738 |
| 46 | α-Linolenic acid | C18 H30 O2 | 96.5 | 21.24 | 279.23148 | 1235925080 |
| 47 | 1-Stearoylglycerol | C21 H42 O4 | 96.4 | 22.83 | 381.29639 | 23515779135 |
| 48 | Naringeninchalcone | C15 H12 O5 | 96.1 | 13.188 | 273.07574 | 175386127.7 |
| 49 | Naringeninchalcone | C15 H12 O5 | 95.3 | 12.293 | 273.07565 | 96402230.64 |
| 50 | 9-Oxo-10(E),12(E)-octadecadienoic acid | C18 H30 O3 | 95.2 | 19.158 | 295.22644 | 1477532221 |
| 51 | Cyclo(phenylalanyl-prolyl) | C14 H16 N2 O2 | 95 | 11.691 | 245.12799 | 105244933.4 |
| 52 | 6,7-dihydro-5H-dibenzo[d,f][1,3]diazepin-6-one | C13 H10 N2 O | 94.7 | 15.499 | 211.08644 | 81775854.29 |
| 53 | Genistein | C15 H10 O5 | 94.4 | 13.017 | 303.08609 | 223515330.5 |
| 54 | DL-Arginine | C6 H14 N4 O2 | 94.3 | 1.1 | 175.11873 | 9685596286 |
| 55 | L-Iditol | C6 H14 O6 | 94.3 | 1.283 | 221.04161 | 1179416927 |
| 56 | trans-3-Indoleacrylic acid | C11 H9 N O2 | 93.8 | 7.16 | 188.07065 | 1140481044 |
| 57 | Anabasine | C10 H14 N2 | 93.8 | 7.726 | 163.12303 | 897747111.6 |
| 58 | (+/-)9,10-dihydroxy-12Z-octadecenoic acid | C18 H34 O4 | 93.8 | 18.2 | 337.23431 | 498608854.7 |
| 59 | 5-hydroxy-2-(4-hydroxyphenyl)-8,8-dimethyl-4H,8H-pyrano[3,2- g]chromen-4-one | C20 H16 O5 | 93.7 | 18.261 | 337.10617 | 2017533184 |
| 60 | Naringenin | C15 H12 O5 | 93.6 | 10.551 | 273.07587 | 146819754.6 |
| 61 | Levallorphan | C19 H25 N O | 93.4 | 13.459 | 284.20068 | 162220503.7 |
| 62 | Asparagine | C4 H8 N2 O3 | 92.9 | 1.521 | 133.06071 | 594944269.7 |
| 63 | 2,2,6,6-Tetramethyl-1-piperidinol (TEMPO) | C9 H19 N O | 92.4 | 15.015 | 158.15384 | 292135106.4 |
| 64 | Dibutyl phthalate | C16 H22 O4 | 92.3 | 18.346 | 279.1586 | 844249844.3 |
| 65 | Phenacetin | C10 H13 N O2 | 92.3 | 1.478 | 180.1017 | 320226448 |
| 66 | 3-Hydroxy-2-methylpyridine | C6 H7 N O | 92 | 9.45 | 110.06041 | 379028038.5 |
| 67 | Caprolactam | C6 H11 N O | 92 | 8.165 | 114.09172 | 179660707.4 |
| 68 | Levallorphan | C19 H25 N O | 91.9 | 12.608 | 284.20084 | 46003483.6 |
| 69 | Vanillin | C8 H8 O3 | 91.4 | 10.368 | 153.05476 | 243941404.1 |
| 70 | N3,N4-Dimethyl-L-arginine | C8 H18 N4 O2 | 91.4 | 1.198 | 203.15018 | 117619081.5 |
| 71 | 4-Aminobenzoic acid | C7 H7 N O2 | 91.3 | 8.668 | 138.05516 | 166526576.6 |
| 72 | (+/-)-Cannabichromeorcin | C17 H22 O2 | 91.2 | 21.983 | 259.16904 | 170427739.3 |
| 73 | 4-Methoxycinnamic acid | C10 H10 O3 | 91.1 | 20.861 | 161.05959 | 416106238.1 |
| 74 | Corymboside | C26 H28 O14 | 90.5 | 12.124 | 565.15491 | 215991534.2 |
| 75 | Phenacetin | C10 H13 N O2 | 88.9 | 12.007 | 180.10194 | 304982221.1 |
| 76 | 5-hydroxy-2-(4-hydroxyphenyl)-8,8-dimethyl-4H,8H-pyrano[3,2- g]chromen-4-one | C20 H16 O5 | 88.8 | 18.092 | 337.10641 | 915516387.4 |
| 77 | 6-Methylquinoline | C10 H9 N | 88.7 | 8.429 | 144.08083 | 300540004.1 |
| 78 | Aflatoxin B2 | C17 H14 O6 | 88.6 | 14.484 | 315.08618 | 139678260.2 |

*(continued)*

Table S1. Continued

| 79 | 12-Oxo phytodienoic acid | C18 H28 O3 | 88.4 | 18.513 | 293.21075 | 803531572.1 |
| --- | --- | --- | --- | --- | --- | --- |
| 80 | (+/-)-Cannabichromeorcin | C17 H22 O2 | 88.1 | 27.113 | 259.16907 | 79710777.7 |
| 81 | Licochalcone A | C21 H22 O4 | 87.9 | 17.621 | 339.15842 | 1852796916 |
| 82 | (1S,4aR,6aS,6bR,10R,11R,12aR)-1,10,11-trihydroxy-9,9-  bis(hydroxymethyl)-2,2,6a,6b,12a-pentamethyl- 1,2,3,4,4a,5,6,6a,6b,7,8,8a,9,10,11,12,12a,12b,13,14b-icosahydropicene- 4a-carboxylic acid | C30 H48 O7 | 87.6 | 15.933 | 521.34686 | 183117047.8 |
| 83 | 6-methyl-4-(morpholinomethyl)-2H-chromen-2-one | C15 H17 N O3 | 86.2 | 7.514 | 260.12817 | 478639060.9 |
| 84 | N-Acetylhistamine | C7 H11 N3 O | 84.8 | 1.084 | 154.09735 | 32260787.08 |
| 85 | N-Acetylhistamine | C7 H11 N3 O | 84.6 | 1.829 | 154.09743 | 633079919.5 |
| 86 | 4-Amino-3-hydroxybenzoic acid | C7 H7 N O3 | 83.9 | 6.806 | 154.04997 | 180470493.7 |
| 87 | N-(2,4-Dimethylphenyl)formamide | C9 H11 N O | 83.7 | 4.876 | 150.09137 | 7235562865 |
| 88 | N-Acetylhistamine | C7 H11 N3 O | 83.3 | 1.414 | 154.09731 | 337147209.5 |
| 89 | Pyridoxal | C8 H9 N O3 | 83.1 | 2.131 | 168.06544 | 973994936.7 |
| 90 | Ursolic acid | C30 H48 O3 | 82.6 | 20.954 | 457.36719 | 198883161 |
| 91 | Argininosuccinic acid | C10 H18 N4 O6 | 81.6 | 1.418 | 291.12949 | 651808597.7 |
| 92 | Guvacoline | C7 H11 N O2 | 80.4 | 5.865 | 142.0864 | 369723005.8 |
| 93 | 1-Linoleoyl glycerol | C21 H38 O4 | 79.8 | 20.127 | 355.28375 | 174492368.3 |
| 94 | 1-(4-nitrophenyl)piperidine | C11 H14 N2 O2 | 79.3 | 6.601 | 207.11287 | 621129400.2 |
| 95 | N-(2,4-Dimethylphenyl)formamide | C9 H11 N O | 78.3 | 12.783 | 150.09155 | 121209781.6 |
| 96 | 2-Amino-1,3,4-octadecanetriol | C18 H39 N O3 | 78.2 | 14.836 | 318.29953 | 539351302.8 |
| 97 | Pyrrole-2-carboxylic acid | C5 H5 N O2 | 78.1 | 2.351 | 112.03961 | 316125576.8 |
| 98 | 3-(2-Hydroxyethyl)indole | C10 H11 N O | 78 | 13.077 | 162.09137 | 367472498.9 |
| 99 | 1,3-Dimethyl-6-morpholino-1,2,3,4-tetrahydropyrimidine-2,4-dione | C10 H15 N3 O3 | 77.8 | 6.284 | 226.1185 | 176371281.6 |
| 100 | Tetramethylpyrazine | C8 H12 N2 | 77.7 | 9.746 | 137.10745 | 99270497.91 |
| 101 | 5-Methoxyindoleacetic acid | C11 H11 N O3 | 77.2 | 8.158 | 206.08119 | 299225444.7 |
| 102 | Resorcinol monoacetate | C8 H8 O3 | 76.9 | 11.665 | 153.0547 | 113422446 |
| 103 | 6-Pentyl-2H-pyran-2-one | C10 H14 O2 | 76.7 | 10.841 | 167.10672 | 122173344.5 |
| 104 | 3-(2-Hydroxyethyl)indole | C10 H11 N O | 76.6 | 13.758 | 162.09126 | 127597109 |
| 105 | Butyl 4-aminobenzoate | C11 H15 N O2 | 76.3 | 13.385 | 194.1174 | 148723218.2 |
| 106 | (3aR,5aR,9bR)-3a-hydroxy-5a,9-dimethyl-3-methylidene- 2H,3H,3aH,4H,5H,5aH,6H,7H,8H,9bH-naphtho[1,2-b]furan-2-one | C15 H20 O3 | 75.2 | 16.578 | 249.14803 | 900617559.7 |
| 107 | 5-hydroxy-4-methoxy-5,6-dihydro-2H-pyran-2-one | C6 H8 O4 | 73.5 | 4.445 | 145.04951 | 9373827640 |
| 108 | Ethylmorphine | C19 H23 N O3 | 73.3 | 7.255 | 314.17493 | 875408737.7 |
| 109 | 1,3-Dimethyl-6-morpholino-1,2,3,4-tetrahydropyrimidine-2,4-dione | C10 H15 N3 O3 | 73.3 | 6.03 | 226.11853 | 108046063 |
| 110 | Nalorphine | C19 H21 N O3 | 72.8 | 8.153 | 312.15952 | 49079319.06 |
| 111 | Codeine | C18 H21 N O3 | 72.3 | 9.695 | 300.15936 | 61072874.02 |
| 112 | Phenacetin | C10 H13 N O2 | 72.2 | 10.428 | 180.10204 | 46701223.64 |
| 113 | 1-[4-(1-adamantyl)phenoxy]-3-piperidinopropan-2-ol hydrochloride | C24 H35 N O2 | 71.3 | 21.869 | 370.27347 | 139837436.4 |
| 114 | 5,7,3',4'-Tetrahydroxy-6,8-diprenylisoflavone | C25 H26 O6 | 71.2 | 19.313 | 423.17938 | 10877495603 |
| 115 | 3-Hydroxy-2-methylpyridine | C6 H7 N O | 71.1 | 10.078 | 110.06056 | 227335266.9 |
| 116 | Ethylmorphine | C19 H23 N O3 | 71.1 | 9.075 | 314.17499 | 421712013.3 |
| 117 | Galangin | C15 H10 O5 | 71 | 13.831 | 271.05991 | 224610193 |
| 118 | Codeine | C18 H21 N O3 | 70.4 | 8.128 | 300.15933 | 114010904.4 |
| 119 | N-Acetyl-DL-tryptophan | C13 H14 N2 O3 | 70.3 | 11.609 | 556.21759 | 721042408.3 |
| 120 | N1-bicyclo[2.2.2]oct-2-ylbenzamide | C15 H19 N O | 70.2 | 17.626 | 230.15364 | 390971186.9 |
| 121 | 6-Methoxyquinoline N-oxide | C10 H9 N O2 | 69.6 | 7.087 | 176.07068 | 169268072.8 |
| 122 | MBZP | C12 H18 N2 | 69.5 | 8.669 | 191.1545 | 681349581.3 |
| 123 | Betulin | C30 H50 O2 | 68.6 | 17.679 | 425.37692 | 1244652370 |
| 124 | 3-(3-pyridinyl)propanoic acid | C8 H9 N O2 | 68.2 | 11.002 | 152.07071 | 216137650.9 |
| 125 | (±)-Abscisic acid | C15 H20 O4 | 68.1 | 15.307 | 265.14316 | 226622246.9 |
| 126 | DL-Stachydrine | C7 H13 N O2 | 68 | 1.79 | 144.10184 | 3643580501 |
| 127 | Methylhippuric acid | C10 H11 N O3 | 68 | 3.359 | 194.08134 | 650818136.9 |
| 128 | 5-Hydroxyindole-3-acetic acid | C10 H9 N O3 | 67.6 | 12.288 | 192.06557 | 121946043.8 |
| 129 | Sorbic acid | C6 H8 O2 | 66.8 | 7.565 | 113.06012 | 199963333.4 |
| 130 | Muramic acid | C9 H17 N O7 | 66.7 | 1.518 | 252.10739 | 281868032.5 |
| 131 | 7-Hydroxycoumarine | C9 H6 O3 | 65.6 | 12.38 | 163.03911 | 129991507.3 |
| 132 | Pentoxifylline | C13 H18 N4 O3 | 65.6 | 7.117 | 279.14526 | 123240394.3 |
| 133 | 4-hydroxy-5,8-dimethylquinoline-3-carboxylic acid | C12 H11 N O3 | 65.1 | 10.462 | 218.08121 | 314124760.4 |
| 134 | 3-(2,3-dihydro-1-benzofuran-5-yl)-1-isopropyldihydro-2,4(1H,3H)- pyrimidinedione | C15 H18 N2 O3 | 65.1 | 7.401 | 275.13907 | 206988059.7 |
| 135 | 3-(2,6-Dioxocyclohexyl)propanenitrile | C9 H11 N O2 | 64.8 | 9.819 | 166.08629 | 589729807.2 |
| 136 | 4-Methoxybenzaldehyde | C8 H8 O2 | 64.5 | 15.595 | 137.05966 | 194698699.9 |
| 137 | 5-amino-2-(dimethylamino)benzoic acid | C9 H12 N2 O2 | 64 | 8.455 | 181.09715 | 320978849.4 |
| 138 | 7-Hydroxycoumarine | C9 H6 O3 | 62.5 | 10.618 | 163.03899 | 139362965.3 |
| 139 | Anabasine | C10 H14 N2 | 62.4 | 6.327 | 163.12303 | 157944533.7 |
| 140 | L-Tyrosine | C9 H11 N O3 | 62 | 1.872 | 182.08121 | 844570011.6 |
| 141 | 4-Methoxybenzaldehyde | C8 H8 O2 | 61.4 | 15.33 | 273.11176 | 438460389.9 |
| 142 | 7-Hydroxycoumarine | C9 H6 O3 | 61 | 9.806 | 163.03899 | 483575575.3 |
| 143 | Dibutylone | C13 H17 N O3 | 60.7 | 11.588 | 236.12813 | 41011245.49 |
| 144 | N-Benzylformamide | C8 H9 N O | 60.6 | 2.782 | 136.07578 | 721619980 |
| 145 | Morphine | C17 H19 N O3 | 60.4 | 7.189 | 286.14368 | 93501822.74 |
| 1 | Linoleic acid | C18 H32 O2 | 100 | 21.891 | 279.23239 | 1890039263 |
| 2 | 16-Hydroxyhexadecanoic acid | C16 H32 O3 | 99.9 | 19.186 | 271.22757 | 84477576.6 |
| 3 | Oleanolic acid | C30 H48 O3 | 99.7 | 21.677 | 455.35239 | 1326261422 |
| 4 | Oleanolic acid | C30 H48 O3 | 99.6 | 22.797 | 455.35236 | 368034929.1 |
| 5 | Stearic acid | C18 H36 O2 | 99.6 | 23.669 | 283.26395 | 105703036.1 |
| 6 | Palmitic Acid | C16 H32 O2 | 99.4 | 22.41 | 255.23236 | 300195878.5 |
| 7 | Nervonic acid | C24 H46 O2 | 99.4 | 27.52 | 365.34167 | 24618303.19 |
| 8 | Oleic Acid | C18 H34 O2 | 97.8 | 22.646 | 281.24808 | 1137170606 |
| 9 | (±)13-HODE | C18 H32 O3 | 97.4 | 19.231 | 295.22723 | 2571567382 |
| 10 | Catechin | C15 H14 O6 | 97.1 | 10.204 | 289.07144 | 501825057.4 |
| 11 | Catechin | C15 H14 O6 | 96.5 | 8.949 | 289.0715 | 337175952.1 |
| 12 | 16-Hydroxyhexadecanoic acid | C16 H32 O3 | 91.1 | 22.387 | 271.22754 | 203431510.4 |

*(continued)*

Table S1. Continued

| 13 | 13(S)-HOTrE | C18 H30 O3 | 89.8 | 18.68 | 293.21185 | 168631284 |
| --- | --- | --- | --- | --- | --- | --- |
| 14 | Thymidine | C10 H14 N2 O5 | 88.9 | 6.373 | 241.08249 | 42271061.8 |
| 15 | (15Z)-9,12,13-Trihydroxy-15-octadecenoic acid | C18 H34 O5 | 88.3 | 16.653 | 329.23264 | 790834850.3 |
| 16 | Caffeic acid | C9 H8 O4 | 86.4 | 10.008 | 179.03397 | 197648164.9 |
| 17 | Naringenin | C15 H12 O5 | 73.1 | 14.335 | 271.061 | 296136953.1 |
| 18 | Naringenin | C15 H12 O5 | 72.2 | 14.72 | 271.06091 | 266324968.5 |
| 19 | Prostaglandin A1 | C20 H32 O4 | 70.8 | 20.102 | 317.21155 | 98308629.53 |
| 20 | α,α-Trehalose | C12 H22 O11 | 63.3 | 1.414 | 387.11346 | 4211720789 |
