# Supplementary material for: Shenling Baizhu San attenuates testicular spermatogenic dysfunction in hyperuricemic mice via dual modulation of MAPK/NF-κB and NLRP3 inflammasome pathways
Source: Hereditas. 2025 Sep 29;162:195. doi: 10.1186/s41065-025-00553-x (PMC12482675; doi:10.1186/s41065-025-00553-x)
Supplement: Supplementary file 2 — Supplementary Material 2 [file 41065_2025_553_MOESM2_ESM.pptx]

## Slide 1
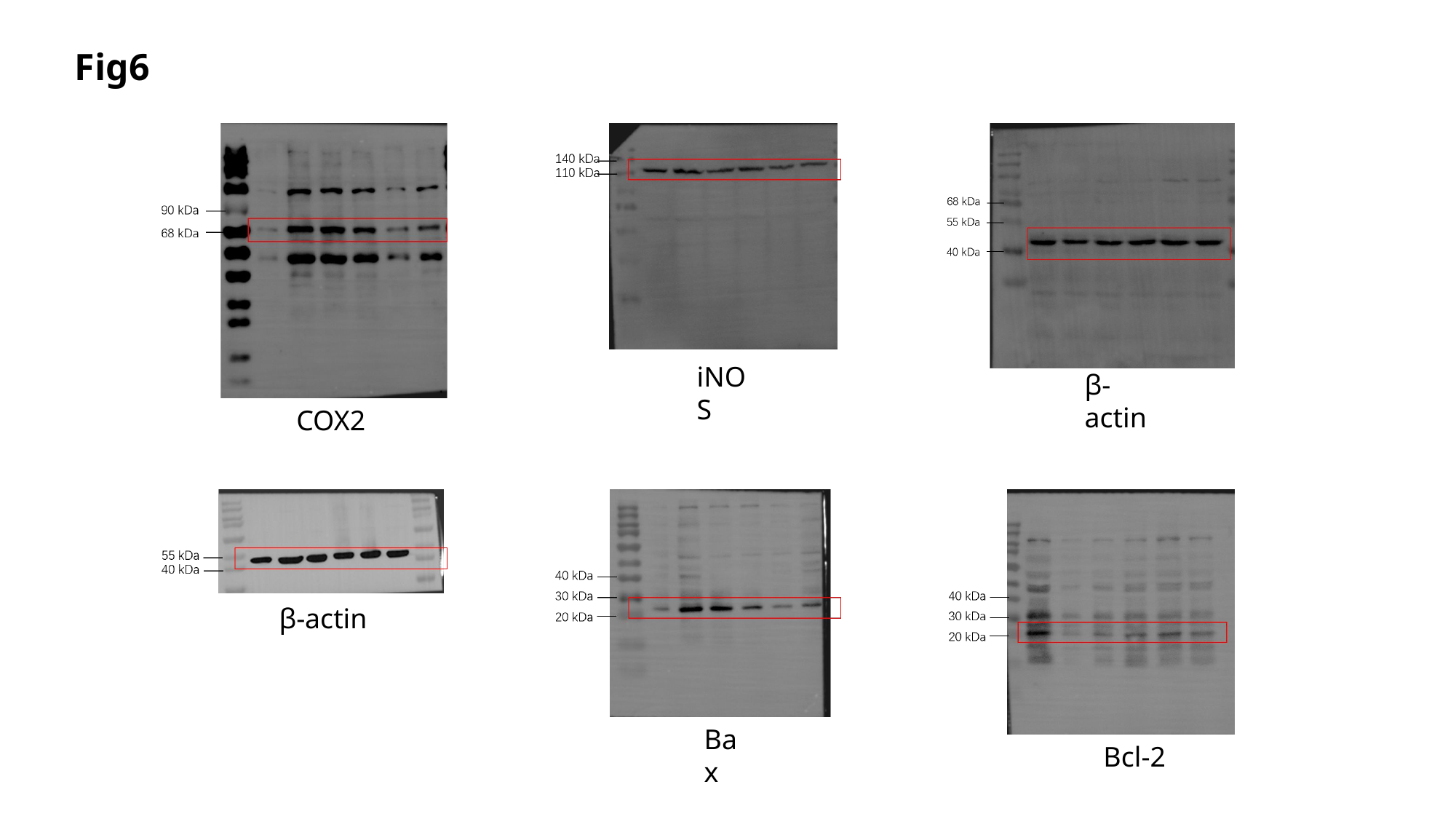

Fig6
iNOS
β-actin
COX2
β-actin
Bax
Bcl-2

## Slide 2
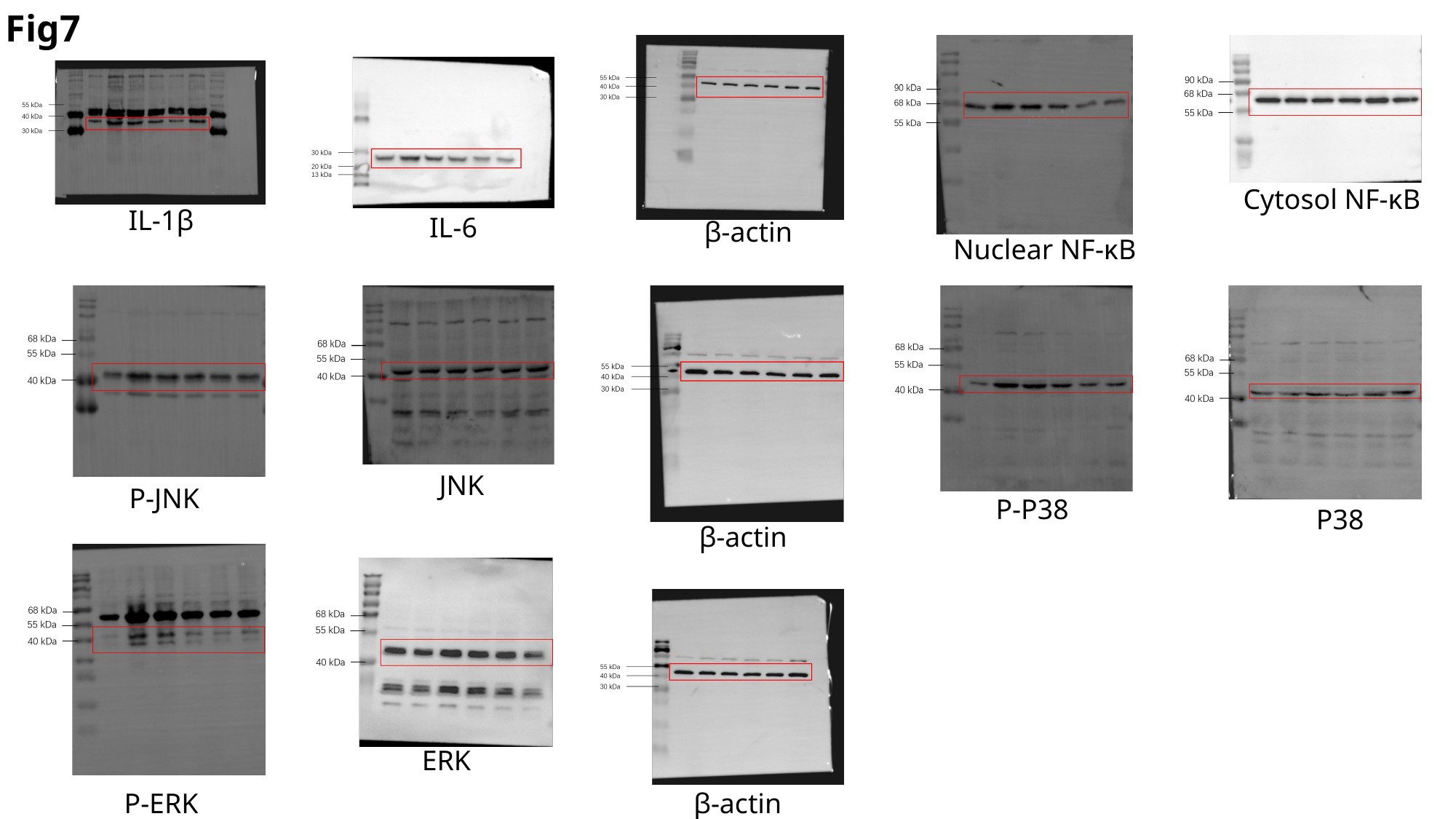

Fig7
Cytosol NF-κB
IL-1β
IL-6
β-actin
Nuclear NF-κB
JNK
P-JNK
P-P38
P38
β-actin
ERK
P-ERK
β-actin

## Slide 3
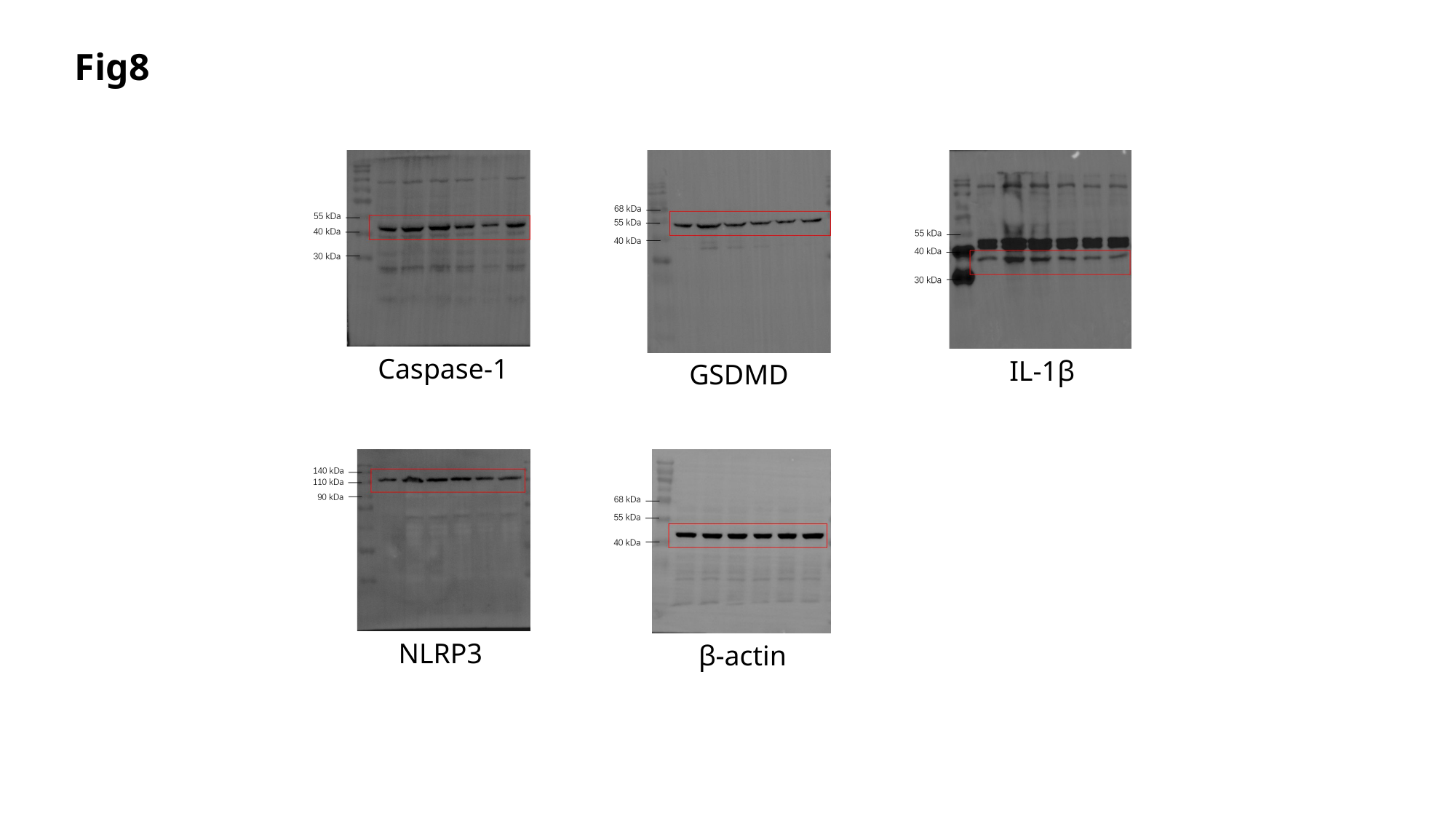

Fig8
Caspase-1
IL-1β
GSDMD
NLRP3
β-actin
